# High throughput cytotoxicity screening of anti-HER2 immunotoxins conjugated with antibody fragments from phage-displayed synthetic antibody libraries

**DOI:** 10.1038/srep31878

**Published:** 2016-08-23

**Authors:** Shin-Chen Hou, Hong-Sen Chen, Hung-Wei Lin, Wei-Ting Chao, Yao-Sheng Chen, Chi-Yu Fu, Chung-Ming Yu, Kai-Fa Huang, Andrew H.-J. Wang, An-Suei Yang

**Affiliations:** 1 Genomics Research Center, Academia Sinica, Taipei 115, Taiwan; 2 Department of Life Science, Tunghai University, Taichung 407, Taiwan; 3 Institute of Cellular and Organismic Biology, Academia Sinica, Taipei 115, Taiwan; 4 Institute of Biological Chemistry, Academia Sinica, Taipei 115, Taiwan

## Abstract

Immunotoxins are an important class of antibody-based therapeutics. The potency of the immunotoxins depends on the antibody fragments as the guiding modules targeting designated molecules on cell surfaces. Phage-displayed synthetic antibody scFv libraries provide abundant antibody fragment candidates as targeting modules for the immunoconjugates, but the discovery of optimally functional immunoconjugates is limited by the scFv-payload conjugation procedure. In this work, cytotoxicity screening of non-covalently assembled immunotoxins was developed in high throughput format to discover highly functional synthetic antibody fragments for delivering toxin payloads. The principles governing the efficiency of the antibodies as targeting modules have been elucidated from large volume of cytotoxicity data: (a) epitope and paratope of the antibody-based targeting module are major determinants for the potency of the immunotoxins; (b) immunotoxins with bivalent antibody-based targeting modules are generally superior in cytotoxic potency to those with corresponding monovalent targeting module; and (c) the potency of the immunotoxins is positively correlated with the densities of the cell surface antigen. These findings suggest that screening against the target cells with a large pool of antibodies from synthetic antibody libraries without the limitations of natural antibody responses can lead to optimal potency and minimal off-target toxicity of the immunoconjugates.

Immunoconjugates are becoming a mainstay in antibody-based therapeutics^1^,^2^,^3^,^4^,^5^,^6^,^7^,^8^,^9^,^10^,^11^,^12^,^13^,^14^,^15^, for which the discovery of antibodies capable of optimally delivering cytotoxic payloads through interacting with cell surface targets is one of the determining steps. The first therapeutic application of protein toxin (diphtheria toxin) was approved by the US Food and Drug Administration in 1999 for Denileukin diftitox in treating cutaneous T-cell lymphoma^12^. Currently, around 10 immunotoxins are under clinical development^11^. About half of the immunotoxins in human trials conjugate with the cytotoxic payload derived from Pseudomonas Exotoxin A (PE)^11^, for which the intoxication mechanism has been well-studied^11^,^16^,^17^. The cytotoxicity of an immunotoxin is effectuated by the antibody-based targeting module inducing receptor-mediated endocytosis^18^, delivering the toxin payload to proper subcellular locations for optimal cytotoxicity. Although antibody-mediated receptor crosslinking^19^ and antibody binding location on the receptor^20^ have been demonstrated as the determinants affecting the efficiency of immunoconjugate-induced endocytosis, discovery of suitable antibodies for delivering cytotoxic payloads through interacting with a specific cell surface target has relied on screening of large number of candidate antibodies^20^,^21^,^22^,^23^,^24^,^25^,^26^—the principles governing the efficiencies for the internalization of the immunoconjugates and the delivery of the toxin payloads remain limitedly understood.

The goal of this study is to elucidate the principles governing the efficiency of the antibodies as targeting modules for cytotoxic drug delivery. Antibodies used as targeting modules in immunoconjugates are more likely to result in optimally functional therapeutics by satisfying the following criteria: adequate affinity and specificity to the target receptor; capable of inducing receptor-mediated endocytosis; capable of delivering the toxin payload to subcellular locations for optimal cytotoxicity; of human origin to reduce immunogenicity; easy to manufacture with high expression efficiency and protein stability. To this end, we have constructed a phage-displayed synthetic antibody library (GH2) with a single human variable domain antibody germline framework: IGKV1-NL1*01/IGHV3-23*04^27^, on which the antibody libraries were designed based on the antibody-protein interaction principles derived from computational and experimental analyses^27^,^28^,^29^,^30^,^31^. On the order of hundreds of antibodies binding to HER2-ECD (human epidermal growth factor receptor 2—extracellular domain) with high affinity and specificity have been discovered from the GH2 library with phage display-based selection and screening^27^. The GH2 antibody epitopes on HER2-ECD are broadly distributed over the HER2-ECD molecular surface and many of the epitopes were novel^27^. Moreover, overwhelming majority of the GH2 antibodies in both scFv and IgG forms can be expressed with high efficiency and high protein stability^27^. As such, the groundwork has been established to explore the applicability of these HER2-ECD-specific GH2 antibodies as targeting modules in corresponding immunotoxins and to elucidate the associated principles governing the efficiency of these antibodies in delivering toxin payloads.

In this work, HER2-overexpressed cells were used as model systems to evaluate efficacies of large number of immunotoxins with diverse antibodies as targeting modules. Because immunotoxin construction rate is limited by the low-throughput recombinant protein production and purification procedures, we developed an adaptor-toxin fusion proteins AL1-PE38KDEL and AL2-PE38KDEL for high throughput screening of the GH2 antibodies as targeting modules for delivering PE38KDEL, which is a truncated form of PE A subunit toxin^17^,^32^. The GH2 library had been constructed with Protein A and Protein L selections so as to ensure that the IGKV1-NL1*01/IGHV3-23*04 framework of the VL and VH domains in a GH2 scFv simultaneously binds to Protein L and Protein A^27^. The AL1 fragment contains consecutive Protein A and Protein L separated by a polypeptide linker enabling the Protein A and Protein L binding to a GH2 scFv simultaneously; the AL2 fragment is composed of two consecutive AL1 modules separated by another polypeptide linker with length designed to promote two GH2 scFvs binding simultaneously to the AL2 fragment. While the scFv-AL1-PE38KDEL is designed as 1:1 for scFv:toxin in one immunoconjugate complex, the scFv-AL2-PE38KDEL is designed to mimic the bivalent antigen binding of IgG with 2 scFvs in one immunoconjugate complex. 92 GH2 scFvs, which bind to HER2-ECD with high specificity and affinity on diverse epitopes, were tested as the targeting modules combining with the AL1-PE38KDEL and AL2-PE38KDEL adaptor-toxin fusion proteins.

The results showed that different scFvs, when conjugate with PE38KDEL, lead to strikingly different cytotoxicity. The bivalent binding promotes superior toxin delivery in some scFvs but shows no obvious difference comparing with monovalent binding in other scFvs. Epitope location on the target receptor determines the efficiency in receptor-mediated endocytosis and/or toxin delivery to certain extent; amino acid types on the antibody paratopes could also be a prominent determinant affecting the subcellular toxin delivery location. The density of the target receptors on the cell surface determines the cytotoxic efficacies of the immunoconjugates as well. These results indicate that the cytotoxicity of the immunotoxins are decisively dependent on the target cells and the antibodies as the targeting modules; screening of a large pool of candidate antibodies as the targeting module is a viable strategy to optimize the antibodies in immunoconjugate developments. While a large pool of candidate antibodies can be difficult to attain from natural antibody repertoires, synthetic antibody libraries can provide functional antibodies without being restricted by natural antibody responses. Combining the synthetic antibody libraries with the high throughput screening platform developed in this work enables better engineering capabilities for developing optimally functional antibody-based targeting modules in immunoconjugates.

## Results

### High throughput screening platform for non-covalently assembled immunotoxins in combination with phage-displayed synthetic antibody libraries

**Figure 1:**
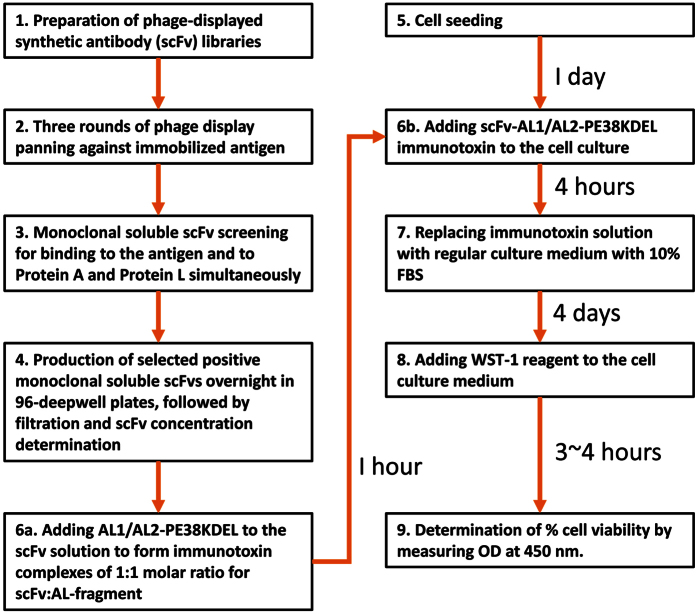
Flow chart of the high throughput screening platform for non-covalently assembled immunotoxins with antibody fragments derived from synthetic antibody libraries. The scFv binders against HER2-ECD were selected in three selection/amplification cycles from the phage-displayed GH2 scFv library (built on IGKV1-NL1*01/IGHV3-23*04 single framework) with the standard phage display selection/screening procedure (Step 1~2)^27^. Candidate scFvs were then screened for soluble scFv binding to HER2-ECD and for binding to Protein A and Protein L (step 3)^27^. Simultaneous binding to Protein A and Protein L indicates that the scFv with the IGKV1-NL1*01/IGHV3-23*04 framework is well-folded (structure shown in PDB code: 4HKZ)^33^. Positive clones binding to HER2-ECD, Protein A, and Protein L were grown in 96-deepwell plates overnight and the culture media were filtrated with 0.45 μm filter (step 4). Cells were seeded in 96-well plates one day ahead in normal medium (10% FBS) (step 5). Soluble scFvs in medium were mixed with AL1/AL2-PE38KDEL in 1:1 (scFv:AL fragment) molar ratio for 1 hour to form the scFv-AL1-PE38KDEL or scFv-AL2-PE38KDEL immunotoxin complexes (step 6a). These complexes are stably formed because of the high affinity scFv binding site in the AL fragment (Figs 2~3) and the preselection of the positive clones with well-folded scFv structure capable of binding to Protein A and Protein L simultaneously. These immunotoxin complexes were added to cultured cells: Normal medium was replaced by 50 μL/well serum free medium before adding 50 μL immunotoxin complex to each well (step 6b). After 4 hours incubation at 37 °C, medium with immunotoxin complex was replaced by normal medium (10% FBS) and the cells were cultured for 4 more days at 37 °C (step 7). WST-1 reagent (10 μL/well) was added to the medium and incubated for 3 to 4 hours for color development (step 8). The cell viabilities were determined at OD_450_ nm by spectrophotometer (step 9). More experimental details are described in Methods.

**Figure 2:**
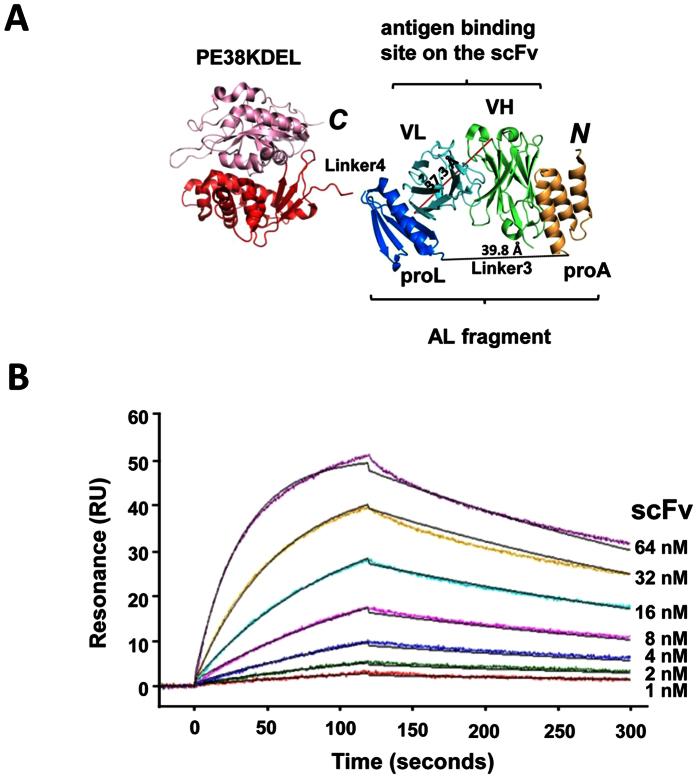
AL1-PE38KDEL adaptor-toxin fusion protein and SPR measurements of AL1-PE38KDEL/scFv interaction. (**A**) The structure of the trastuzumab scFv variable domain (VH in green and VL in cyan) in complex with protein A (orange) and protein L (blue) is derived from PDB code: 4HKZ. The distance between the *C*-terminal residue of the VL domain and the *N*-terminal residue of the VH domain is shown by the red dotted line; the distance between the *C*-terminal residue of the Protein A and the *N*-terminal residue of the protein L is shown by the black dotted line. The PE38KDEL, a truncated form of *Pseudomonas* exotoxin (PDB code: 1IKQ), is colored in red and pink for domain II and III respectively. (**B**) SPR measurements of immobilized AL1-PE38KDEL passed over with 1, 2, 4, 8, 16, 32 and 64 nM Transtuzumab scFv are shown with representative binding traces in colors; the overlaying black lines are the global fit to the experimental data with K_*d*_ = 5.64 ± 0.03 × 10^−9^ M; k_*on*_ = 4.54 ± 0.06 × 10^5^ M^−1^S^−1^ and k_*off*_ = 2.56 ± 0.04 × 10^−3 ^S^−1^ (see Methods).

**Figure 3:**
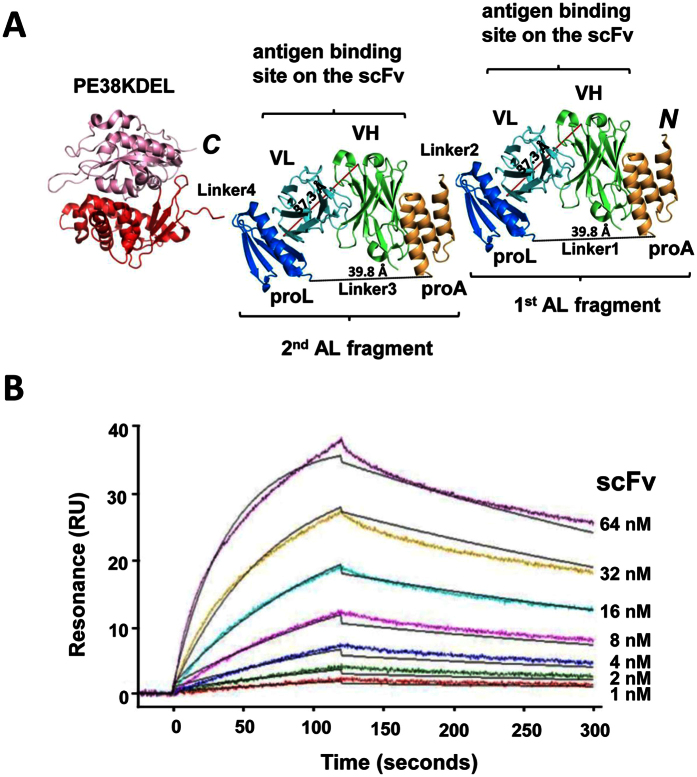
AL2-PE38KDEL adaptor-toxin fusion protein and SPR measurements of AL2-PE38KDEL/scFv interaction. (**A**) The AL2-PE38KDEL adaptor-toxin fusion protein has one additional AL fragment linked by a 5-residue linker (Linker2) to the N-terminus of the AL1-PE38KDEL as shown in Fig. 2A. (**B**) SPR measurements of immobilized AL2-PE38KDEL interacting with soluble scFv. The description is the same as in Fig. 2B. The overlaying black lines are the global fit to the experimental data with K_*d*_ = 5.52 ± 0.03 × 10^−9 ^M; k_*on*_ = 3.60 ± 0.06 × 10^5 ^M^−1^S^−1^ and k_*off*_ = 1.99 ± 0.04 × 10^−3 ^S^−1^.

High throughput screening tests a large number of monoclonal antibodies for their biological function in parallel. Conventional tests of the function of immunotoxins require labor- and material-intensive sub-cloning and purification of the immunotoxins, limiting the throughput rate in discovering and optimizing the targeting antibodies. Monoclonal GH2 synthetic antibodies can be consistently expressed to the concentration on the order of 10 nM to 100 nM in soluble scFv form secreted by host *E. coli* cells into about 1 ml of medium in 96-deepwell plates^27^. To enable a high throughput screening platform for the soluble scFvs in culture media without purification of the scFvs (Fig. 1), we constructed the adaptor-toxin fusion proteins: AL1-PE38KDEL (Fig. 2) and AL2-PE38KDEL (Fig. 3), according to the structure of Protein A and Protein L in complex with a scFv of IGKV1-NL1*01/IGHV3-23*04 framework (PDB code: 4HKZ)^33^ (Figs 2A and 3A). These adaptor-toxin fusion proteins binds to recombinant scFv or IgG with the IGKV1-NL1*01/IGHV3-23*04 framework, self-assembling into non-covalently-linked immunotoxin complexes in crude mixture of culture media, allowing the self-assembled immunotoxin complexes to be tested on cells cultures in micro-titer plates for cytotoxicity assay in high throughput format without the rate-limiting processes of subcloning and purification (Fig. 1).

### Non-covalently assembled immunotoxins with monovalent targeting modules

AL1-PE38KDEL binds to scFv of IGKV1-NL1*01/IGHV3-23*04 framework in high affinity. The N-to-C sequential arrangement of the Protein A and Protein L in the AL fragment of the adaptor-toxin fusion protein AL1-PE38KDEL (Fig. 2A) is essential to avoid the clash between the Linker3 and the VL-VH linker, which is a 15-residue polypeptide linking the N-to-C sequential arrangement of the VL and VH domains in the scFv construct of the GH2 library (Fig. 2A). Linker3 is a 15-residue polypeptide linker (SG_4_)_3_, providing enough length to accommodate a scFv molecule simultaneously binding to the Protein A and the Protein L in the AL fragment. Linker4 is a 5-residue linker (G_2_SG_2_) connecting the AL fragment to the toxin PE38KDEL. The adaptor-toxin fusion protein AL1-PE38KDEL has one high affinity scFv binding site, enabling binding of Protein A to the VH domain and Protein L to the VL domain simultaneously (Fig. 2A). Alternative binding configurations with only one Protein A/L interacting with the scFv molecule are of relatively low affinity with dissociation constant K_*d*_ on the order of 10^−6^~10^−7 ^M. Hence these monovalent complex configurations are not expected to be significantly populated in the scFv-AL1-PE38KDEL complexes. This anticipation has been validated with the binding affinity and binding kinetics of scFv to AL1-PE38KDEL measured with surface plasmon resonance (SPR) (see Methods), from which the results indicated K_*d*_ = 5.64 ± 0.03 × 10^−9 ^M with k_*on*_ = 4.54 ± 0.06 × 10^5 ^M^−1^S^−1^ and k_*off*_ = 2.56 ± 0.04 × 10^−3 ^S^−1^ (Fig. 2B).

### Non-covalently assembled immunotoxins with bivalent targeting modules

The adaptor-toxin fusion protein AL2-PE38KDEL is expected to bind to two scFvs of IGKV1-NL1*01/IGHV3-23*04 framework with two consecutive AL fragments. The two AL fragments are linked by Linker2, which is a 5-residue linker (G_2_SG_2_) to prevent the formation of a scFv-binding site composed of the Protein L from the first AL fragment and the Protein A from the second AL fragment (Fig. 3A). As such, the AL2-PE38KDEL adaptor-toxin fusion protein is expected to contain two independent scFv binding sites with nano-molar affinity. Although the complex configuration binding to one scFv could occur by forming the complex with the Protein A from the first AL fragment and the Protein L from the second AL fragment, this configuration is anticipated to have lower affinity to the scFv in comparison with the binding configuration shown in Fig. 3A. This anticipation arises on the basis that the entropy cost of forming the complex fixing the N- and C-termini encompassing the long intervening fragments (the Protein L and Protein A from the first and the second AL fragment respectively) is much higher than that of forming per scFv-AL complex as shown in Fig. 3A. This anticipation has been validated by the measurement of the binding affinity and binding kinetics of scFv to AL2-PE38KDEL (see Methods) with surface plasmon resonance: K_*d*_ = 5.52 ± 0.03 × 10^−9 ^M with k_*on*_ = 3.60 ± 0.06 × 10^5 ^M^−1^S^−1^ and k_*off*_ = 1.99 ± 0.04 × 10^−3 ^S^−1^ (Fig. 3B). These measurements are similar to those for scFv binding to AL1-PE38KDEL (Fig. 2B), indicating that the AL2-PE38KDEL has two independent scFv-binding sites, each of which has binding thermodynamics and kinetics similar to those of the AL fragment in the AL1-PE38KDEL; the complex configuration shown in Fig. 3A is expected to be the main state of the complex configurations for the scFv-AL2-PE38KDEL complexes.

### AL-scFv complex formation does not interfere with scFv-HER2-ECD interaction

**Figure 4:**
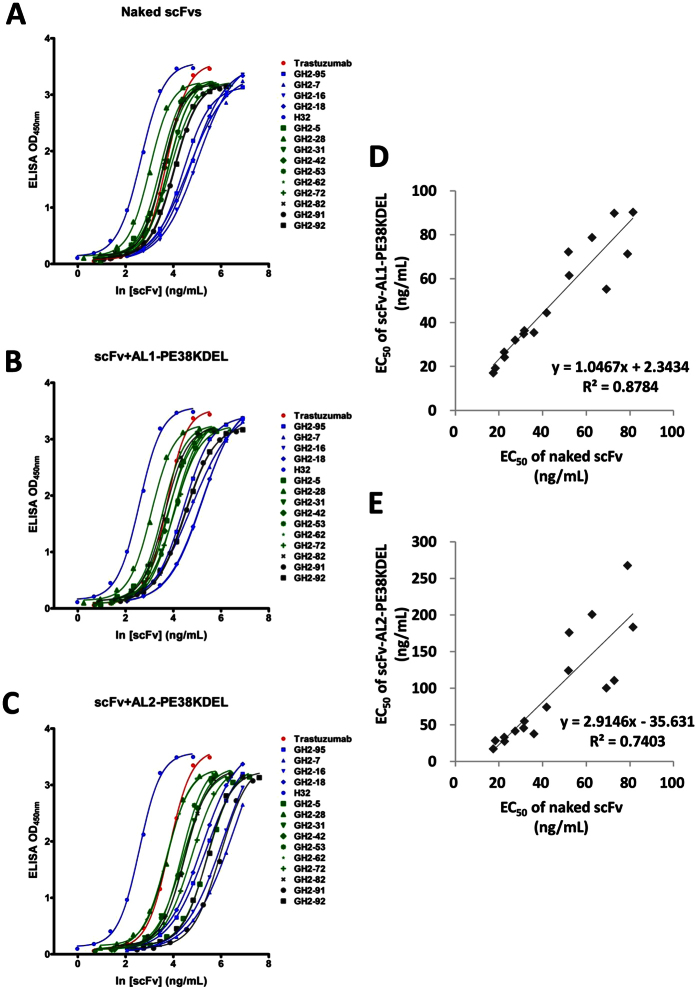
Binding affinities of scFvs in the presence and absence of AL1/AL2-PE38KDEL. (**A**) 16 scFvs with IGKV1-NL1*01/IGHV3-23*04 germline framework were used to measure the EC_50_ for the scFv-HER2-ECD binding. (**B**) Same set of scFvs as in panel (**A**) were mixed with AL1-PE38KDEL with 1:1 molar ratio of the scFv:AL fragment, and the EC_50_’s for the scFv-HER2-ECD binding were measured. (**C**) Same description as in panel (**B**) for AL2-PE38KDEL. (**D**) The HER2-ECD-binding EC_50_’s for the scFv-AL1-PE38KDEL complexes (from panel (**B**)) are plotted versus those for the naked scFvs (from panel (**A**)). The x- and y-axis show the EC_50_ in concentration (ng/mL) of the scFv. The EC_50_ values for the scFvs are listed in Supplementary Table S1. (**E**) Same description as in panel (**D**) for AL2-PE38KDEL.

Protein A and Protein L binding to a scFv of the IGKV1-NL1*01/IGHV3-23*04 framework has been known to have little impact on the binding of the scFv to its corresponding antige^29^,^30^. Quantitative comparisons of the scFv-HER2-ECD binding affinities in the presence and absence of AL1-PE38KDEL and AL2-PE38KDEL further validate this conclusion (Fig. 4). 16 positive monoclonal scFvs were randomly selected from the GH2 library binding to HER2-ECD and binding to Protein A and Protein L simultaneously (Fig. 1, step 1~step 4). The EC_50_’s for the scFv-HER2-ECD binding were measured in the absence (Fig. 4A) and presence of AL1-PE38KDEL (Fig. 4B) or AL2-PE38KDEL (Fig. 4C) (data shown in Supplementary Table S1). The formation of the stable scFv-AL1/AL2-PE38KDEL complexes had been evident by ensuring that the scFvs bound to Protein A and Protein L simultaneously with the established procedure^27^. Plotting HER2-ECD-binding EC_50_’s for the scFv-AL1-PE38KDEL complexes versus those for the same set of ‘naked’ scFvs shows slope of 1.0 and R^2^ = 0.88 (Pearson’s correlation coefficient = 0.94) (Fig. 4D), indicating that quantitatively, the binding of AL1-PE38KDEL to each of the 16 scFvs does not affect the corresponding scFv-HER2-ECD interaction. Plotting HER2-ECD-binding EC_50_’s for the scFv-AL2-PE38KDEL complexes versus those for the ‘naked’ scFvs shows slope of 2.9 and R^2^ = 0.74 (Pearson’s correlation coefficient = 0.86) (Fig. 4E), indicating that the effective concentration of the scFv in the scFv-AL2-PE38KDEL system was reduced by three folds, perhaps because the scFv-AL2-PE38KDEL complexes are less effective to generate the equivalent ELISA signal strength comparing with the corresponding ‘naked’ scFv or scFv-AL1-PE38KDEL complex. The high linear correlations between the affinities of the ‘naked’ scFvs with those of the scFvs in the scFv-AL1-PE38KDEL (Fig. 4D) or scFv-AL2-PE38KDEL (Fig. 4E) complexes indicate that the complex formation does not affect the relative affinities of the scFvs binding to the antigen molecule.

### Non-covalently assembled scFv-AL1-PE38KDEL and scFv-AL2-PE38KDEL can be potent immunotoxins with subnano-molar IC_50_’s

**Figure 5:**
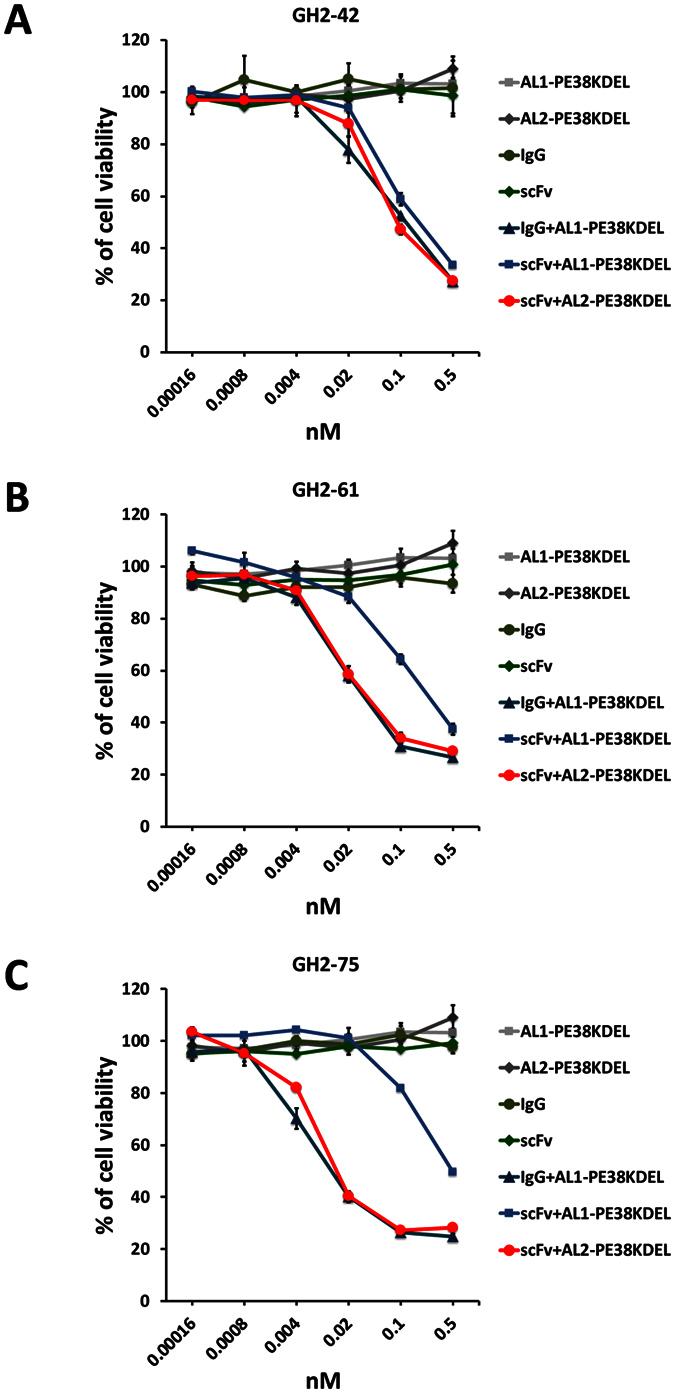
Cytotoxicity of scFv or IgG complexed with the adaptor-toxin fusion protein AL1-PE38KDEL or AL2-PE38KDEL. N87 cells were treated with controls (AL1-PE38KDEL, AL2-PE38KDEL, scFv, and IgG) or non-covalently assembled immunotoxins (IgG-AL1-PE38KDEL, scFv-AL1-PE38KDEL, and scFv-AL2-PE38KDEL), for which the cytotoxicity was determined by the procedure shown in Fig. 1. (**A**) Cytotoxicity test results for the antibody GH2-42 in scFv or IgG form. The y-axis of the plots shows the percentage of cell viability of the treated N87 cells (see Methods); the x-axis shows the concentration of the VL-VH variable domain—the antibody (IgG or scFv) and the adaptor-toxin fusion protein (AL1-PE38KDEL or AL2-PE38KDEL) were mixed in 1:1 molar ratio for VL-VH:AL fragment. The error bars were determined with three independent repeats of the measurements. (**B**) Cytotoxicity test results for the antibody GH2-61 in scFv or IgG form. (**C**) Cytotoxicity test results for the antibody GH2-75 in scFv or IgG form. The description for panels (**B**,**C**) is the same as in panel (**A**).

Both types of scFv-AL2-PE38KDEL and scFv-AL1-PE38KDEL complexes are potent immunotoxins. IgGs with the IGKV1-NL1*01/IGHV3-23*04 framework for the VL and VH variable domains complexed with AL1-PE38KDEL were comparable immunotoxins as the corresponding scFv-AL2-PE38KDEL (Fig. 5). The scFv(GH2-42)-AL1-PE38KDEL, scFv(GH2-61)-AL1-PE38KDEL, and scFv(GH2-75)-AL1-PE38KDEL are similar in potency as immunotoxins, with IC_50_ ≅ 0.1 nM—the concentration of the scFv-AL1-PE38KDEL at which 50% of the cells tested were killed by the immunotoxin (Fig. 5A~C). The IgG-AL1-PE38KDEL immunotoxins are equally potent as the corresponding scFv-AL2-PE38KDEL complexes for the three representative antibodies (Fig. 5A~C), most likely because these two types of immunotoxins have bivalent antibody targeting modules. The immunotoxins with bivalent targeting modules are more cytotoxic comparing with the corresponding immunotoxins with monovalent targeting modules (Fig. 5A~C); the extent of the bivalent effect varies depending on the details of the antibody-antigen interactions (Fig. 5A~C). The results suggest that although the scFv-AL2-PE38KDEL complexes reduce the effective concentration of scFv in ELISA measurements (Fig. 4E), the cell-based cytotoxicity measurements nevertheless indicate the superior potency of the immunotoxins with bivalent antigen binding sites. The ELISA measurements were not indicative for the potency of the immunotoxins; direct measurements of cell-based cytotoxicity are essential for screening the potency of the immunotoxins. Overall, the technical platform depicted in Fig. 1 is feasible for high-throughput screening of scFv/IgG as targeting modules in non-covalently assembled immunotoxins with mono- or bi-valent targeting modules.

### High throughput screening of non-covalently assembled immunotoxins

**Figure 6:**
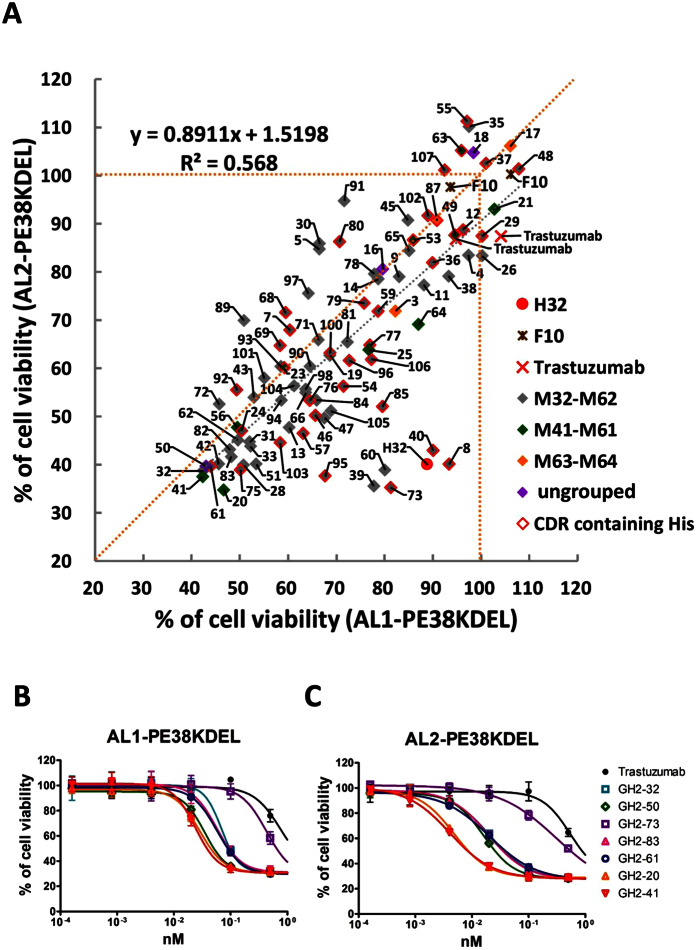
Cytotoxicity screening of 92 HER2-ECD-specific scFv-AL1/AL2-PE38KDEL immunotoxins. (**A**) The percentages of cell viability for N87 cells treated with scFv-AL1-PE38KDEL or scFv-AL2-PE38KDEL were determined following the procedure depicted in Fig. 1 and are shown in the x-axis and y-axis respectively. The soluble scFv in culture medium was adjusted to concentration of 0.5 nM in the presence of corresponding concentration of AL1-PE38KDEL (x-axis) or AL2-PE38KDEL (y-axis) for 1:1 molar ratio of scFv:AL fragment. Four epitope groups of these GH2 scFvs are marked in gray (M32-M62), green (M41-M61), orange (M63-M64), and purple (ungrouped). GH2 scFvs with histidine(s) in CDR-H3 are indicated by the red frame over the symbol. The detailed data for each of the scFvs are listed in Supplementary Table S2. (**B**) Selected GH2 scFvs were subjected to IC_50_ measurements for the scFv-AL1-PE38KDEL complexes. The y-axis shows the percentage of cell viability of the treated N87 cells and the x-axis shows the concentration of the scFv (1:1 molar ratio of scFv:AL fragment). The error bars were calculated with three independent repeats of the measurements. (**C**) Same description as in panel (**B**) for the scFv-AL2-PE38KDEL complexes.

92 HER2-ECD-specific scFvs selected from the GH2 synthetic antibody library^27^ were tested as the targeting modules in the non-covalently assembled immunotoxins with the high throughput screening platform (Fig. 1). The CDR sequences and the associated data of this set of scFvs are listed in Supplementary Table S2. The soluble scFv in culture medium was adjusted to concentration of 0.5 nM in the presence of corresponding concentration of AL1-PE38KDEL or AL2-PE38KDEL for 1:1 molar ratio of scFv:AL fragment. The immunotoxin solutions were applied to test the cytotoxicity in micro-titer plate (Fig. 1); the cell viability readouts showed wide-range of cytotoxicity of the immunotoxins at the concentration of 0.5 nM of scFv for both types of immunotoxins (Fig. 6A). The IC_50_’s of a few of the most potent immunotoxins were determined by cytotoxicity measurements for cells treated with serial dilutions of the scFv-AL1-PE38KDEL (Fig. 6B) and scFv-AL2-PE38KDEL immunotoxins (Fig. 6C). The GH2-41 and GH2-20 scFvs appear as the most effective targeting modules from the set of the 92 scFvs in both form of immunotoxins, and the bivalent form is superior to the monovalent form in terms of cytotoxicity by about 10 folds, with IC_50_ < 10 pico-molar. By comparison, trastuzumab scFv as a targeting module is about 100 folds inferior in terms of IC_50_ to the best scFvs in the set of scFvs tested (Fig. 6B,C).

The epitopes and binding affinity/kinetics of the tested scFvs are not the major determinants for the potency of the immunotoxins. The cytotoxicity of the scFv-AL1-PE38KDEL immunotoxins is largely linearly correlated with the cytotoxicity of the corresponding scFv-AL2-PE38KDEL immunotoxins with slope = 0.89 and R^2^ = 0.57 (Pearson’s correlation coefficient = 0.75) (Fig. 6A). A slope less than one in the plot indicates that bivalent immunotoxins are in general more potent than single valent immunotoxins; the bivalent effect is dependent on the details of the scFv-antigen interaction (Fig. 5), explaining the scattering of the data points in the plot. Moderate linear correlation in the plot suggests that intrinsic properties of the scFv-HER2 interaction determine the potency of the mono- and bivalent immunotoxins. However, the predominant determinants for the potency of the immunotoxins are not obvious: scFvs from the same epitope group (color coded as shown in the data points in Fig. 6A, see also Supplementary Table S2) have wide range of cytotoxicity, suggesting that the cytotoxicity of the immunotoxins depends on parameters not limited by the epitope locations on the antigen surface. EC_50_, K_*d*_, k_*on*_, and k_*off*_ for the scFvs plotted against the cytotoxicity of the corresponding scFv-AL1-PE38KDEL immunotoxins (Supplementary Figure S1) and the cytotoxicity of the corresponding scFv-AL2-PE38KDEL immunotoxins (Supplementary Figure S2) show correlation coefficient close to zero, indicating that these binding affinity and binding kinetics parameters of the scFvs are not predominant determinants for the immunotoxins’ potency.

The amino acid types on the CDR regions of the scFvs could be an important determinant affecting the cytotoxicity of the immunotoxins. Only CDRH3 of the GH2 synthetic scFvs are distributed with ionizable residue His (Supplementary Table S2, see also the data points with red outline in Fig. 6A), which might affect the cytotoxic potency of the immunotoxins because the His sidechain is expected to change charge state (pKa = 6.5) when the immunotoxins are internalized from environment of pH 7 to expose to increasingly acidified environment with the pH approaching 5 in late endosomes. If the CDRH3 contains His residues and the His residues are involved in antibody-antigen interactions, these His residues could determine the subcellular locations where the immunotoxins dissociated from the target receptors at acidic pH. This dissociation location could decide if the immunotoxins get transferred to cytosol. We divide the 92 scFvs into 4 quarters according to the scFv-AL1-PE38KDEL cytotoxicity (Supplementary Table S2) and find that the ratios for scFvs containing His in CDRH3 are 6/23, 8/23, 10/23, and 13/23 respectively for the four quarters of the set of 92 scFv-AL1-PE38KDEL immunotoxins with decreasing cytotoxicity (Fig. 6A and Supplementary Table S2). This monotonic trend suggests that the His sidechains involving antibody-antigen interactions could be a determinant for the toxin payload trafficking routes, which in turn determine the cytotoxicity of the immunotoxins.

To test the effect of CDR His sidechains in determining the cytotoxicity of the immunotoxins, we measured the pH-dependency of the immunotoxin-HER2 binding affinities for 4 subsets of scFv-AL1-PE38KDEL with diverse cytotoxicities and His residue distributions in the CDR regions. We randomly grouped 4 subsets of scFv-AL1-PE38KDEL from the set of immunotoxins belonging to the M32-M62 epitope group in Fig. 6A (grey diamond data points with red outline (His in CDR) or without red outline (no His in CDR)): the first set scFv-AL1-PE38KDEL (GH2-42, 72, 62, 31, 101) are among the top 25% in terms of cytotoxicity and without His residue in the CDR regions (Supplementary Figure S3A); the second set (GH2-61, 92, 75, 24, 103) are also among the top 25% in terms of cytotoxicity but with His residue(s) in the CDR regions (Supplementary Figure S3B); the third set (GH2-11, 38, 4, 26, and H32) are among the bottom 25% in terms of cytotoxicity and without His residue in the CDR regions (Supplementary Figure S3C); the fourth set (GH2-102, 40, 107, 8, 12, 55) are also among the bottom 25% in terms of cytotoxicity and with His residue(s) in the CDR regions (Supplementary Figure S3D). Only the fourth group contains members for which the HER2-ECD affinities are substantially compromised at pH5 in comparison with those at pH7 (Supplementary Figure S3 and Supplementary Table S3). The first and the third groups do not contain His residue in the CDR regions and thus the pH-independency of the affinities to their antigen is as expected. The pH-independency of the affinities of the second group (Supplementary Figure 3B) and the pH-dependency of the affinities of the fourth group (Supplementary Figure 3D) indicate that the interruption of the antibody-antigen interactions due to ionization of His sidechains at acidic environment of endosome could be one of the factors attributing to the low cytotoxicity of the immunotoxins, in agreement with the conclusion in the previous paragraph. The affinity of scFv(trastuzumab)-AL1-PE38KDEL to HER-ECD is high and pH-independent (Supplementary Figure S3C) and thus the low cytotoxicity of this immunotoxin is most likely attributed to the epitope of trastuzumab on HER2-ECD domain IV, which is distant to the M32-M62 epitope group on domain I of HER2-ECD.

### Cell surface target receptor expression level determines the potency of the immunotoxins

**Figure 7:**
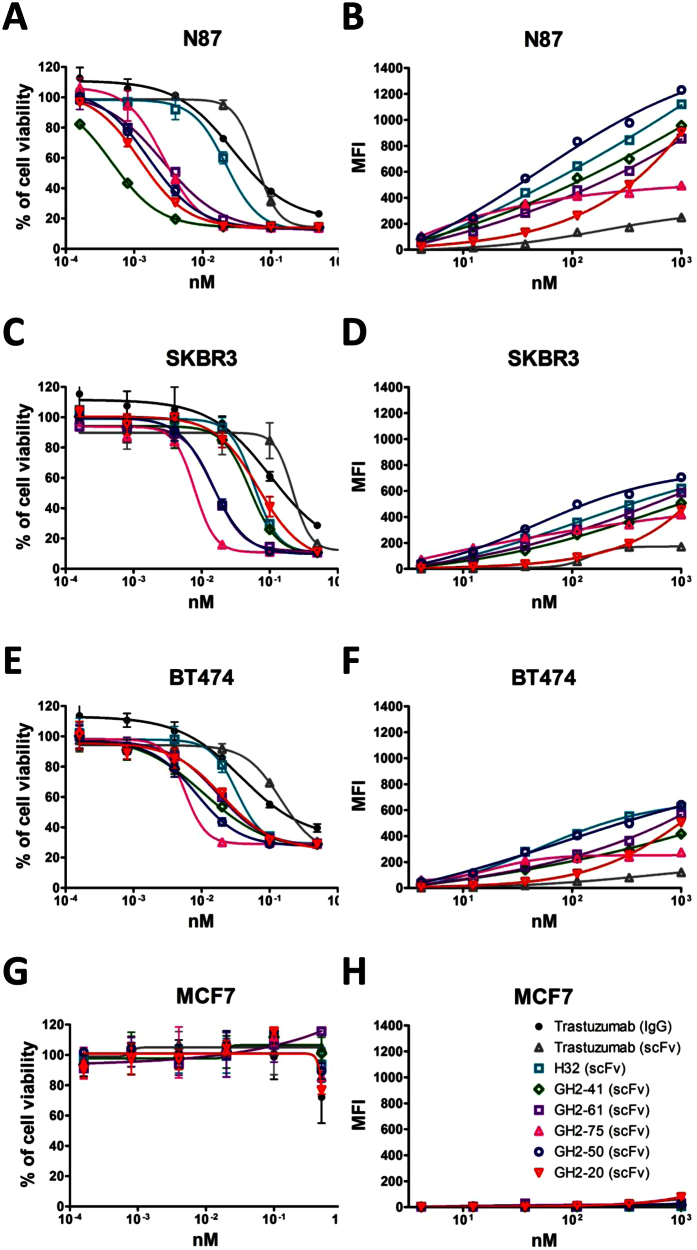
Cytotoxicity and mean fluorescence intensity of immunotoxins on cells with different HER2 expression level. (**A**,**C**,**E**,**G**) The IC_50_’s for N87 (panel (**A**)), SKBR3 (panel (**C**)), BT474 (panel (**E**)) and MCF7 (panel (**G**)) cells were measured with selected GH2 scFvs complexed with AL2-PE38KDEL in 1:1 ratio of the scFv:AL fragment. The y-axis shows percentage of cell viability (Methods); the x-axis shows the concentration of scFv. The error bars were calculated with three independent repeats of the measurements. (**B**,**D**,**F**,**H**) Mean fluorescence intensities (MFIs) for the same set of scFvs complexed with AL2-RFP (see Methods) in 1:1 ratio of the scFv:AL fragment were measured with N87 (panel (**B**)), SKBR3 (panel (**D**)), BT474 (panel (**F**)), and MCF7 (panel (**H**)) cells by flow cytometry, where the MFIs shown in the y-axis were adjusted over the background value by the cells treated with AL2-RFP without scFv. The x-axis shows the concentration of the scFv.

The distribution density of the target receptor HER2 (Fig. 7B,D,F,H) affects the cytotoxicity of the immunotoxins (Fig. 7A,C,E,G). The N87 cell line has the highest HER2 receptor density on the cell surface (Fig. 7B) among the cell lines tested in Fig. 7, and the immunotoxins (in scFv-AL2-PE38KDEL form) are the most potent, with the lowest IC_50_’s for this cell line (Fig. 7A). The SKBR3 and BT474 cell lines have less HER2 receptor density (Fig. 7D,F) in comparison with that of N87; the cytotoxicity of the corresponding immunotoxins measured with these cell lines are also less potent (Fig. 7C,E). In the negative control cell line MCF7, where only negligible density of HER2 shows on the cell surface (Fig. 7H), the cells are not sensitive to the immunotoxins (Fig. 7G). Note that the affinity ranking of the scFvs to the cell surface receptor is not correlated with the ranking of the cytotoxicity of the immunotoxin (Fig. 7). Moreover, the potency ranks of the scFv as targeting modules are also dependent on the cell line tested (Fig. 7A,C,E), indicating that differences between cells could affect the cytotoxicity of the immunotoxins.

### Antibody binding to HER2-ECD on cell surface leads to endocytic trafficking of the antibody to late endosome and Golgi complex

**Figure 8:**
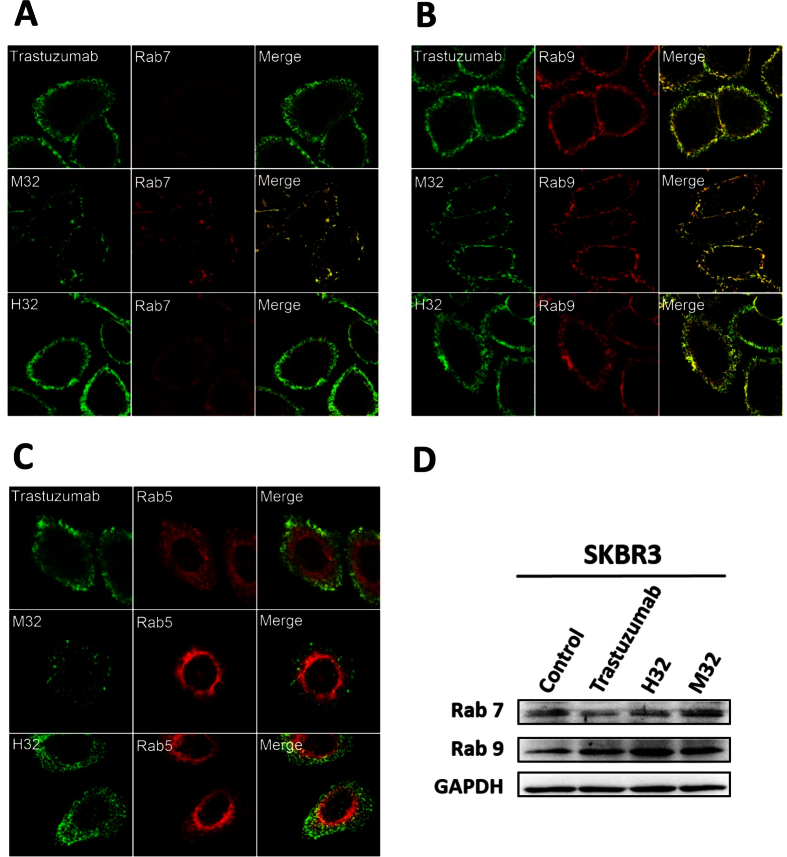
Comparison of the internalization pathways of M32, H32, and Trastuzumab IgGs binding to HER2-ECD on cell surface. (**A**) SKBR-3 cells were incubated with trastuzumab, M32 or H32 IgG for 1 hour, and the localizations of the internalized IgGs in relation to those of Rab7 were revealed by immunofluorescence microscopy. (**B**) Same description as in panel (**A**) for Rab9. (**C**) Same description as in panel (**A**) for Rab5. (**D**) Relative expression levels of Rab7 and Rab9 in the SKBR-3 cells treated with trastuzumab, M32 or H32 IgG were analyzed by Western blot, where GAPDH was detected as internal control. The Western blots were derived under the same experimental conditions from the same cell lysates; the original full-length Western blot images are showed in Supplementary Figure S4. The expression levels were reproducible with three independent experimental repeats.

The endocytic mechanism of the antibody-HER2 complexes was further investigated by following the antibody (H32, M32, and trastuzumab) internalization locations in relation to the Rab5, Rab7, and Rab9 GTPases (Fig. 8). Antibody H32 is the humanized version of antibody M32^27^, which is a control mouse anti-HER2 antibody, for which the epitope on HER2-ECD is representative for the majority of the antibodies in Fig. 6A; more than three quarters of the 92 scFvs in Supplementary Table S2 belong to this epitope group (dubbed M32-M62 epitope group in Supplementary Table S2)^27^. The internalization of H32 IgG upon binding to HER2 is 1.4 fold more efficient than that of trastuzumab based on the biotinylation assay (see Methods), explaining in part the superior potency of H32 comparing with trastuzumab as targeting modules in immunotoxins (Fig. 6A). Figure 8 shows that the trafficking routes for these HER2-binding IgGs are identical in terms of their subcellular locations relative to Rab5, Rab7 and Rab9. Rab5 is associated with early endocytic pathway and Rab7 and Rab9 with the late endocytic pathway—Rab7 and Rab9 are located around late endosomes^34^. Co-localization of the antibody M32, H32, and trastuzumab with Rab7 (Fig. 8A) and Rab9 (Fig. 8B) but not with Rab5 (Fig. 8C) suggests that the antibodies were internalized following the late endocytic pathway after binding to HER2-ECD on cell surface. The antibody-associated receptor endocytosis also enhanced expression of Rab9, especially by antibody H32 (Fig. 8D and Supplementary Figure S4). Rab9 has been known to regulate the recycling of mannose-6-phosphate receptor from late endosomes to the Golgi complex^35^,^36^. The better-enhanced Rab9 expression due to antibody H32 binding to HER2 could explain the high potency of scFv(H32)-AL2-PE38KDEL, for which the potency as an immunotoxin is much higher than that of scFv(trastuzumab)-AL2-PE38KDEL (Fig. 6A). The enhanced expression of Rab9 also supports the notion that the trafficking of the endocytic antibody-associated receptors could be driven to the Golgi complex following the retrograde trafficking pathway.

### Fab H32 binds to domain I in HER2-ECD

**Figure 9:**
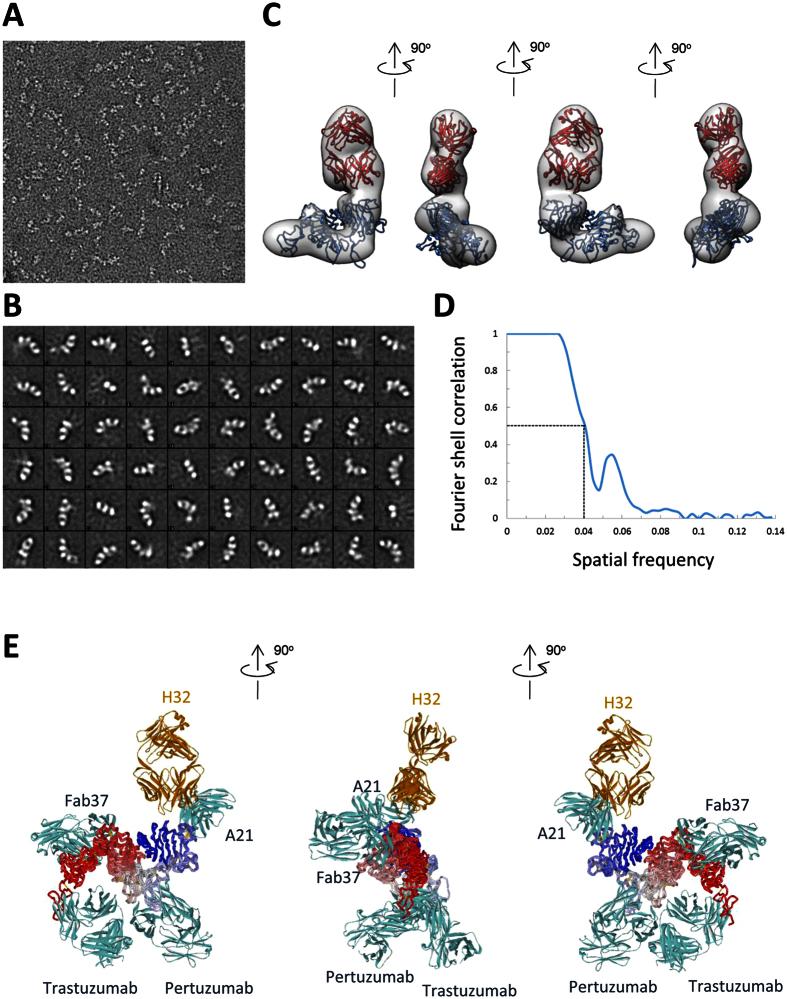
Negative stain EM reconstruction of Fab(H32)-HER2-ECD complexes. (**A**) Negative stain EM of HER2 ECD- Fab(H32) complexes are visible in a representative micrograph. (**B**) Representative 2D class averages are analyzed by the ISAC software (Methods). (**C**) 3D model of HER2 ECD- Fab(H32) complexes (in gray) are reconstructed by the Relion software (Methods). Model structures of Fab(H32) (colored in red) and HER2-ECD (colored in blue, PDB code: 3WSQ) were fit to the 3D molecular envelop with resolution of 23 Å using the UCSF chimera software. (**D**) Gold standard FSC curve of the 3D model with 0.5 cutoff marked by dash lines is shown in the plot. Details of the EM structure determination are described in Methods. (**E**) The complex model of Fab(H32) (colored in golden yellow) with HER2-ECD (colored in blue-to-red for the N-to-C alpha-carbon trace) determined by EM are superimposed with the crystallographic structures of A21 (scFv, PDB code: 3H3B), pertuzumab (Fab, PDB code: 1S78), Fab37 (Fab, PDB code: 3N85) and trastuzumab (Fab, PDB code: 1N8Z). The superimposed HER2-ECDs from each of the PDB complex structures are colored in blue-to-red for the N-to-C alpha-carbon traces. The orientation of the superimposed composite structure shown in this panel is identical to that of the left EM molecular envelope in panel (**C**), where the geometrical arrangement of the HER2 domains I~IV and the relative location of the Fab(H32) can be unambiguously identified.

Fab H32-HER2-ECD complexes were analyzed by negative stain EM and single particle reconstruction (Fig. 9A). 2D class averages suggested that the specimens were relatively homogeneous in form of 1:1 antibody-antigen complex (Fig. 9B). Domains of Fab H32 can be clearly identified in some 2D class averages depending on their orientations. Domains of HER2-ECD can as well be identified in some 2D class averages viewing from the sides where domain I, II, III, and smaller tail domain IV can be assigned. The densities of HER2-ECD, especially domain IV, in some 2D classes were less clear than those of Fab H32, suggesting structural flexibility and variations among HER2-ECD domain relative locations. Particles of selected 2D classes were subsequently subjected to 3D reconstruction and refined to 23 Å resolution with gold standard FSC = 0.5 cutoff (Fig. 9C,D). The 3D map was fit with model Fab and the crystallographic structure of HER2-ECD (Fig. 9C). The data suggests that Fab H32 interacts with HER2-ECD through region next to the epitope of A21 in domain I (Fig. 9E), which agree well with the analysis of 2D projection images (Fig. 9C), the HDX-MS and the competition data (Fig. 6 of Chen *et al*.^27^). The epitope of H32 is different from any observed epitopes on HER2 (Fig. 9E). The epitope difference between the antibody H32 and trastuzumab could explain the potency difference of the immunotoxins based on these two antibodies.

## Discussion

The work highlights three aspects in developing an optimally functional immunotoxin. First, immunotoxins with bivalent antibody-based targeting module are generally superior to those with corresponding monovalent targeting module in term of cytotoxic potency by up to 10 folds (Figs 5~6), although most of the immunotoxins currently under development adopt monovalent design^11^. Bivalent targeting modules could result in cross-linked cell surface receptors, which could in turn accelerate receptor-mediated endocytosis and decrease the off rate of the immunotoxins. Both could assist the trafficking of the toxin to cytosol. Similar results on HER2^37^ and TF^38^ (tissue factor) specific antibody-drug conjugates (ADC) have been reported by comparing the ADC efficacy on cultured cells in IgG and Fab form. However, the data also suggest that the bivalent enhancement of immunotoxin cytotoxicity is antibody-dependent, i.e. the bivalent binding promotes superior toxin delivery in some scFvs but shows no obvious difference comparing with monovalent binding in other scFvs (Figs 5 and 6). Similar conclusion has also been derived from the study of antibody-mediated internalization of human CD73 on cancer cells^39^. Consequently, since prediction of the potency of a immunotoxin based on the valence, epitope and binding affinity of the antibody targeting module is unlikely to be accurate, experimental tests in high throughput format as described in this work can provide substantial information in optimizing the functional immunotoxins.

Second, the potency of the immunotoxins is positively correlated with the densities of the cell surface antigen (Fig. 7). But the correlation level could range from a few folds to more than 100 folds loss of potency (e.g. antibody GH2-41, see Fig. 7) due to ~2 folds reduction of cell surface target density, perhaps because of other differences among the cells. The implication is that the immunotoxins could be engineered to have optimal cytotoxicity towards the target cells with abundant cell surface targets while remain tolerable in terms of toxicity against off-target cells with moderately lower level of target density on the cell surface.

Third, epitope of the antibody-based targeting module is a major determinant for the potency of the immunotoxins (Figs 8~9). However, antibodies in the same epitope group can have diverse potency in delivering the toxin payloads (Fig. 6). Other factors, especially the amino acid types used in the paratope of the antibody, could determine the trafficking route of the toxin payload and thus affect the potency of the immunotoxin. Engineering pH-dependency of antibody-antigen interactions has demonstrated that ionization of engineered His sidechains of the antibody-antigen interface in the acidic environment of endosome enhances cycling of antibody back to plasma, improving pharmacokinetics and duration of the antibody^40^,^41^. By contrast, the results shown in Fig. 6A, Supplementary Figure S3 and Supplementary Table S3 indicate that the ionization of the His sidechains involving antibody-antigen interactions in the increasingly acidic environment of endosome could interrupt the immunotoxin trafficking, compromising the cytotoxicity of the toxin payloads.

The results of this work reveal the complex function of the antibody as the targeting module in an immunotoxin, highlighting the need of multifaceted optimization of the toxin delivery capability of the targeting antibody according to the specifics of the target receptor, the target cell, and the cytotoxic payload. Natural antibodies have been the main source for the targeting modules in immunotoxins, but the diversity of the natural antibodies is restricted by predominant clonal selections governed by germline gene usage and by clonal elimination resulting from immune-tolerance of self-antigens, which are frequently the targets of immunotoxins. As such, natural antibodies as the targeting modules for the immunotoxins could be suboptimal because of limited pool of suitable candidates from natural antibody repertoires. By contrast, properly designed and constructed synthetic antibody libraries could provide more diverse selections of antibodies with epitope-paratope combinations better-suited for developing immunotoxins or other types immunoconjugates against self-antigens with optimal cytotoxicity. The work herein provides the evidence supporting the implications of high throughput discovering and optimizing antibodies as the targeting modules for immunoconjugates with the synthetic antibody libraries.

The future perspectives of this work are twofold: first, determination of the therapeutic properties, including pharmacokinetics and antitumor efficacy, of the selected antibody candidates requires reformation of the antibodies with covalently conjugated toxin payloads for *in vivo* experiments on animal models; second, optimization of immunoconjugates with the candidate antibodies conjugated with diverse toxin payloads derived from natural toxin proteins or small molecular weight cytotoxins through chemical linkers of different chemical activities can be explored with the prototypical high throughput development model established in this work. The noncovalent immunotoxin complexes in this work are not anticipated for *in vivo* experiments. The reason is that the high concentration of IgGs in animal blood could compromise the stability of the scFv-AL1 complexes, resulting in premature dissociation of the noncovalent immunotoxin complexes due to the interaction of AL1 fragment with the IgGs in animal blood. Instead, recombinant fusion proteins with antibody component covalently linked with protein toxin are more suitable for the follow-up *in vivo* preclinical tests and in clinical trials. The data from this work would help the design of the immunoconjugates by finalizing the antibody fragment selection. Moreover, the procedure established in this immunoconjugate development model can be extended to developing antibody-drug conjugates (ADCs), where the optimization procedure needs to consider even larger number of options of chemical toxin payloads and chemical linkers in combination with antibody-based targeting modules. The prototypical high throughput development model for immunoconjugates described in this work can be adapted to effectively screen for ADCs with optimal efficacy on respective disease model systems *in vitro*.

## Methods

### Cell lines

N87, SKBR3, BT474, and MCF7 cells were obtained from the American Type Culture Collection (ATCC). N87 and SKBR3 cells were cultured in RPMI 1640 (Gibco) with 10% fetal bovine serum (Gibco) and penicillin-streptomycin (100X; Gibco). BT474cells were cultured in Hybri-Care Medium (ATCC) with 10% fetal bovine serum (Gibco) and Penicillin/Streptomycin/Glutamine (100X; Gibco). MCF7 cells were cultured in RPMI 1640 (Gibco) with 10% fetal bovine serum (Gibco) and penicillin-streptomycin (100X; Gibco). Suspension HEK293 Freestyle (293-F, Life Technologies, USA) cells were grown in serum free Freestyle 293 expression media (Gibco) at 37 °C shaken with 110 rpm in 8% CO_2_ incubator (Thermo Scientific).

### AL1-PE38KDEL and AL2-PE38KDEL gene construction and protein expression/purification

AL1-PE38KDEL and AL2-PE38KDEL genes based on the published structure of Protein A/L fragments (PDB code: 4HKZ) and a truncated form of *Pseudomonas aeruginosa* Exotoxin A^17^,^32^ (PDB code: 1IKQ) were synthesized as described in Figs 2A and 3A. For plasmid construction, the synthesized genes were sub-cloned into pET32a expression vector (Novagen) encoding a *N*-terminal thioredoxin fusion protein to the AL1-/AL2-PE38KDEL via *Sfi*I and *Not*I restriction sites, and the resultant fusion protein contains a hexa-His tag C-terminal to the thioredoxin, followed by a TEV protease cutting site before the AL1-/AL2-PE38KDEL.

For protein expression, the constructed plasmids were transformed into *E. coli* Rosetta-gami B (DE3) strain (Novagen), and selected colonies were grown in 2 × YT medium (Tryptone 16 g/L, Yeast extract 10 g/L, NaCl 5 g/L) with ampicillin (200 μg/L), tetracycline (12.5 μg/L) chloramphenicol (37.5 μg/L) and kanamycin (15 μg/L) at 37 °C until cell density of OD at 600 nm reached 1.0, and were then incubated at 16 °C for another 1 hr. The cultures were induced with 0.5 mM IPTG overnight at 16 °C. After centrifugation, the harvested cells were re-suspended in buffer A (500 mM NaCl and 5 mM imidazole in 20 mM Tris-HCl, pH 8.0), lysed by Microfluidizer (Microfluidics, MA), and then centrifuged at 40,000 × *g* for 1 hr. The supernatant was loaded onto a HisTrap HP column (GE Healthcare) equilibrated with buffer A. After washing with 30 mL buffer A, the column was eluted by a linear gradient of 0–50% buffer B (the same recipe as that in A, except for 1000 mM imidazole). The eluted AL1-/AL2-PE38KDEL fractions at 0.14–0.24 M imidazole were pooled and digested with His-tagged TEV protease (A_280_ ratio 50∶1) at 30 °C for 3 hr, and then dialyzed against buffer A. The cleaved thioredoxin and TEV protease, both containing hexa-His tag, were removed by HisTrap HP column. The AL1-/AL2-PE38KDEL proteins in the flow-through were collected and further purified with a Superdex 200 size-exclusion column (GE Healthcare) in SEC buffer (150 mM NaCl in 50 mM Tris-HCl, pH 8.0,). Isolated proteins were further analyzed by SDS-PAGE to check their purity and molecular mass.

### Gene construction and protein expression/purification of AL2-RFP/GFP

The expression and purification of the AL2-RFP followed the standard *E. coli* expression and purification methods. In brief, the coding region of AL2-GGGSG-RFP/GFP (red fluorescence protein or green fluorescence protein) with a 6 × His-tag at N-terminus was codon-optimized for *E. coli* expression and cloned into pET-32b expression vector. Cultures of *E. coli* BL21 (DE3) strain (Merck) transformed with the AL2-RFP/GFP construct in pET32b expression vector were grown in 2 × YT medium (Tryptone 16 g/L, Yeast extract 10 g/L, NaCl 5 g/L) with ampicillin (150 μg/L) at 37 °C until OD at 600 nm reached 1.0~2.0, and then incubated at 16 °C for another 1hr before adding 0.5 mM IPTG. After overnight (at least 16 hr) expression, the cells were centrifuged and the pellets were re-suspended in lysis buffer (Tris-HCl 20 mM, NaCl 150 mM, imidazole 10 mM, pH 8.0) and the suspended cells were then lysed by Microfluidizer (Microfluidics, MA). The recombinant AL2-RFP/GFP fusion proteins were purified by Ni^2+^-IMAC column (GE Healthcare Life Sciences) with imidazole gradient from 10 mM to 500 mM in TS solution (Tris-HCl 20 mM, NaCl 150 mM, pH 8.0). The AL2-RFP/GFP fusion protein was further purified by Superdex200 size-exclusion column (GE Healthcare Life Sciences) in PBS buffer and stored at −80 °C. The purified protein was analyzed by SDS-PAGE and coomassie blue staining.

### scFv expression/purification

For preparation of secreted scFv, ER2738 *E. coli* cells were infected with phage harboring the phagemid pCANTAB5E containing the scFv gene for 30 min. The infected ER2738 cells were amplified in 2 × YT with 100 μg/mL ampicillin for 5–6 hr before adding 10 mM IPTG. After overnight culture, the culture supernatant containing the secreted soluble scFv was filtrated by 0.22 μm filter.

For preparing the purified trastuzumab scFv with E-tag, the gene sequence derived from trastuzumab Fab (PDB code: 4HKZ) was synthesized and constructed by connecting VL-VH variable domains with a linker polypeptide, (G)_4_S(G)_4_S(G)_4_S, and introducing the E-tag oligopeptide (GAPVPYPDPLEPRAA) to the C-terminal end. The resulting gene was then cloned into pET32a expression vector (Novagen) via *SfiI* and *NotI* restriction sites. The detailed procedures of scFv protein expression and purification were followed as previously described^31^.

### Concentration determination of scFv in culture medium

The concentration of the secreted scFv culture medium was determined by comparing the ELISA signal to purified standard scFv of known concentration. In brief, Protein L (0.2 μg per well) was coated in PBS buffer (pH 7.4) on NUNC 96-well Maxisorb immuno-plates overnight at 4 °C, and blocked with 5% skim milk in PBST [0.05% (v/v) Tween 20] for 1 hr. Secreted scFvs and standard scFv were serially diluted with culture medium 2 × YT. After blocking, 50 μL diluted samples were added to each well with 50 μL 5% skim milk in PBST [0.05% (v/v) Tween 20], and incubated for 1 hr under gentle shaking. The plate was washed 6 times with 300 μL PBST and then added with 100 μL 1:20000-diluted horse-radish peroxidase/anti-E-tag IgG antibody conjugate (ICL) in PBST with 5% milk for 1 hr incubation. The plates were washed six times with PBST buffer and twice with PBS, developed for 3 min with 3,3′,5,5′-tetramethyl-benzidine peroxidase substrate (Kirkegaard & Perry Laboratories), quenched with 1.0 M HCl and read spectrophotometrically at 450 nm. Concentrations of secreted scFvs were determined with the standard curve derived with scFv of known concentration.

### Surface plasmon resonance (SPR) measurement of binding kinetics of scFv to AL1-PE38KDEL and AL2-PE38KDEL

BIAcore T200 (GE Healthcare) instrument was used to determine the binding affinities and kinetics parameters between scFv and AL1-/AL2-PE38KDEL. Purified AL1-/AL2-PE38KDEL protein in 10 mM acetate buffer (pH 4.5) was immobilized on a CM5 sensor chip to a response unit (RU) of 250–300 with an amine coupling kit. Association (k_*on*_) and dissociation (k_*off*_) constants of the interactions between AL1-/AL2-PE38KDEL and scFv were measured in HBS-EP+ running buffer (10 mM HEPES pH 7.4, 150 mM NaCl, 3 mM EDTA, 0.05% v/v Surfactant P20; GE Healthcare) with a flow rate of 30 μL/min. The sensor surface was regenerated with 10 mM Glycine, pH 2.0, prior to a new scFv injection and the signals obtained were subtracted by that obtained from the reference channel, which had not been coated with adaptor-toxin fusion protein. Binding kinetics was determined by global fitting to 1:1 binding model using the Biaevaluation software (GE Healthcare). Each measurement was confirmed in triple repeats.

### Measurements of EC_50_ with ELISA for scFv binding to AL1-PE38KDEL and AL2-PE38KDEL

For determining the EC_50_ of soluble scFv binding to AL1-/AL2-PE38KDEL, ELISA assay was carried out following the published methods with some modifications^27^. In brief, the HER2/ECD antigen (0.3 μg per well) was coated in PBS buffer (pH 7.4) on NUNC 96-well Maxisorb immunoplates overnight at 4 °C, and blocked with 5% skim milk in PBST [0.05% (v/v) Tween 20] for 1 hr. In the meantime, the secreted scFv in filtered supernatant was mixed with the AL1-/AL2-PE38KDEL at a molar ratio of 1:1 for scFv:AL fragment. After 1 hr incubation at room temperature, the scFv or scFv-AL1-/AL2-PE38KDEL mixtures were prepared at 11 concentrations by twofold serial dilutions with 2 × YT culture medium. 50 μL of diluted scFv samples were mixed with 50 μL of blocking buffer and then added to the blocked microtiter plate for another 1 hr under gently shaking. The plates were washed 6 times with 300 μL PBST (when measuring EC_50_ at pH 7 or pH 5, the wells were soaked in PBST with precise pH (7 or 5) for 5 minutes between the third and the fourth PBST wash), and then added with 100 μL 1: 4000-diluted horse-radish peroxidase/anti-E tag antibody conjugate (Abcam) in PBST with 5% milk for 1 hr incubation. The plates were washed six times with PBST buffer and twice with PBS, developed for 2 min with 3,3′,5,5′-tetramethyl-benzidine peroxidase substrate (Kirkegaard & Perry Laboratories), quenched with 1.0 M HCl and read spectrophotometrically at 450 nm. The EC_50_ (ng/mL) was calculated according to Stewart and Watson method^42^.

### Cytotoxicity measurements for AL1-PE38KDEL and AL2-PE38KDEL

The procedure for determining the cytotoxic effects of immunotoxins is shown schematically in Fig. 1. In brief, 10^4^ cells/well were first seeded in 96-well plates. IgGs or scFvs were pre-incubated with AL1-PE38KDEL/AL2-PE38KDEL at a molar ratio of 1:1 for scFv:AL fragment for 1 hr at room temperature. This procedure allows formation of non-covalently linked immunotoxins. After serially dilution in culture medium 2 × YT, scFv-AL1/AL2-PE38KDEL mixtures were added to cell culture without serum. Culture medium 2 × YT with AL1-PE38KDEL or AL2-PE38KDEL were used as negative control. After 4 hours of incubation at 37 °C, the antibody-toxin mixture was replaced by fresh normal medium with serum. After 4 days of culture at 37 °C, the cells were treated with WST-1 (Roche) following the manufacturer’s instruction; the percentage of cell viability was quantified by calculating the ratio below:


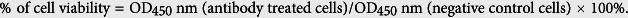


### Mean fluorescence intensity measurements of scFv with AL2-RFP on cell surface by Flowcytometry

Binding of scFv on HER2-expressed cells was measured by FACS. Cells were suspended by trypsin treatment and went through mesh of 40 micron pore. About 2 × 10^5^ cells were incubated with 100 μL scFv at 4 °C for 30 minutes, then washed once with 0.5% FBS 1 × PBS (wash buffer), mixed with 1.2 μg AL2-RFP in 50 μL wash buffer at 4 °C for 20 minutes, and then washed twice with washer buffer. After centrifugation and resuspension, cells were analyzed for RFP signal by FACS (BD FACS Canto II).

### Confocal Microscopy for IgG internalization

SKBR-3 Cells grown on glass coverslips were incubated with 1 μg/mL trastuzumab, M32 or H32 IgG at 37 °C for 1 hr. After washing, cells were fixed with 3.7% formaldehyde and permeabilized with 0.1% Triton-X 100. The fixed cells were incubated with rabbit anti-Rab5, Rab7 or Rab9 antibody (1:200 dilution in PBS/0.1% Triton-100/3% BSA) at room temperature for 1 hr and then incubated with Cy3-conjugated anti-rabbit secondary antibodies (1:200 dilution in PBS/0.1% Triton-100/3% BSA) at room temperature for 1 hr. The presence of trastuzumab, M32 or H32 IgG was revealed by Cy2-conjugated anti-human secondary antibody. Coverslips were mounted with Gel Mount aqueous mounting medium (Sigma, St. Louis, MO, USA). Images were acquired using a Zeiss LSM 510 META confocal microscope with a 63× objective (1.4 oil).

### Detection of IgG induced vesicle protein expressions by Western blot

SKBR-3 cells that had been treated with 1 μg/mL trastuzumab, M32 or H32 IgG at 37 °C for 1 hour were scraped in lysis buffer (1% NP-40, 50 mM Tris pH 7.4, 150 mM NaCl, 2 mM MgCl_2_, 1 mM EGTA, and protease and phosphatase inhibitors). Cellular protein from lysates was separated by SDS–PAGE, and then transferred to PVDF membrane for Western blot detection with antibodies against Rab7 and Rab9, and GAPDH, followed by incubation with appropriate HRP-conjugated secondary antibodies. Blots were developed using an enhanced chemiluminescence system.

### Antibody internalization by biotinylation assay

SKBR-3 cell membranes were biotinylated using EZ-link Sulfo-NHS-SS-Biotin (Pierce, IL) in PBS at 4 °C for 30 min. Labeled cells were washed twice in cold PBS and then incubated with 10 μg/mL trastuzumab or H32 IgG at 37 °C for internalization, after 1 hr, biotin groups remaining on the cell surface were cleaved off at 4 °C with reducing buffer (100 mM sodium-2-mercaptoethane sulfonate, 50 mM Tris, pH 8.6, 100 mM NaCl). The cells were then washed three times in cold PBS and scraped in lysis buffer. Biotinylated membrane complex of HER2 receptor and internalized antibody were captured on streptavidin ELISA plates (Nunc immobilizer; Nunc,Roskilde, Denmark) from the cell lysates diluted to 10 μg/mL total protein in PBS for 1 hr of incubation at room temperature. The plates were then washed three times with PBS and incubated with horseradish peroxidase (HRP)-conjugated anti-human secondary antibody for 1 hr and washed three times in PBS, and the HRP signal was revealed by incubation with OPD color substrate (Sigma, St. Louis, MO). Color development was analyzed at 492 nm using an ELISA reader.

### Negative stain EM and image processing

The antibody-antigen complexes at approximately 8 μg/mL were applied to glow-discharged, carbon-coated copper grids. The grids were washed and stained with 0.75% (wt/vol) uranyl formate for 30 sec. The specimens were imaged with a FEI Tecnai F20 electron microscope operated at 120 kV. Micrographs were recorded with a Gatan UltraScan 1000 CCD at 1.21 Å/pixel and at an average defocus of 1.3 μm. The images were corrected for contrast transfer function, and determined the defocus value using CTFfind3^43^. The projections of the particles were boxed using DoG picker^44^ in Appion^45^, and classified to 2D classes at 3.63 Å/pixel using the software package ISAC^46^ and Relion^47^. The 3D initial models were generated by EMAN2 *ab initial* common line method^48^. The initial models were refined with approximately 60,000 particles using Relion^47^ to 23 Å resolution with gold standard FSC = 0.5 cutoff. Crystal structures of Fab and HER2 ECD (PDB 3wsq) were fit to the 3D model to map the binding region using UCSF chimera^49^.

### Other experimental details

Reformation of scFv into IgG and the expression and purification procedures of IgGs have been published^27^ and are described in details in Supplementary Methods.

## Additional Information

**How to cite this article**: Hou, S.-C. *et al*. High throughput cytotoxicity screening of anti-HER2 immunotoxins conjugated with antibody fragments from phage-displayed synthetic antibody libraries. *Sci. Rep.* **6**, 31878; doi: 10.1038/srep31878 (2016).

## Supplementary Material

Supplementary Information
