# Supplementary material for: High throughput cytotoxicity screening of anti-HER2 immunotoxins conjugated with antibody fragments from phage-displayed synthetic antibody libraries
Source: Sci Rep. 2016 Aug 23;6:31878. doi: 10.1038/srep31878 (PMC4994030; doi:10.1038/srep31878)
Supplement: Supplementary Information [file srep31878-s1.doc]

**Supplemental Information**

**High throughput cytotoxicity screening of anti-HER2 immunotoxins conjugated with antibody fragments from phage-displayed synthetic antibody libraries**

Shin-Chen Hou^a,b^, Hong-Sen Chen^a,b^, Hung-Wei Lin^c^, Wei-Ting Chao^c^, Yao-Sheng Chen^d^, Chi-Yu Fu^d^, Chung-Ming Yu^a^, Kai-Fa Huang^e^, Andrew H.-J. Wang^e*^, An-Suei Yang^a,*^

^a^ Genomics Research Center, Academia Sinica, Taipei, Taiwan 115.

^b^ These authors contribute equally.

^c^ Department of Life Science, Tunghai University, Taichung, Taiwan 407.

^d^ Institute of Cellular and Organismic Biology, Academia Sinica, Taipei, Taiwan 115.

^e^ Institute of Biological Chemistry, Academia Sinica, Taipei, Taiwan 115.

^*^ Correspondence should be addressed to: An-Suei Yang, Genomics Research Center, Academia Sinica, 128 Academia Rd., Sec.2, Nankang Dist., Taipei, Taiwan 115. Phone: +886-2-2787-1232 email: [yangas@gate.sinica.edu.tw](mailto:yangas@gate.sinica.edu.tw); Andrew H.-J. Wang, Institute of Biological Chemistry, Academia Sinica, 128 Academia Rd., Sec.2, Nankang Dist., Taipei, Taiwan 115. Phone: +886-2-2788-1981 email: ahjwang@gate.sinica.edu.tw

**Running title**: screening synthetic antibodies for immunotoxins

**Keywords**: synthetic antibody library, phage display, high throughput screening, immunoconjugate, immunotoxin

**Inventory of Supplemental Information**

**Supplementary Figures**

**Supplementary Figure S1. EC_50_, K_D_, k_on_, and k_off_ vs. cytotoxicity of scFv-AL1-PE38KDEL immunotoxins.**

**Supplementary Figure S2. EC_50_, K_D_, k_on_, and k_off_ vs. cytotoxicity of scFv-AL2-PE38KDEL immunotoxins.**

**Supplementary Figure S3. Measurements of EC_50_’s of scFv-AL1-PE38KDEL at pH 7 and pH 5.**

**Supplementary Figure S4. Western blot of Rab7 and Rab9 from antibody (IgG) treated SKBR-3 cells.**

**Supplementary Tables**

**Supplementary Table S1. Related to Figure 4. EC_50_’s for selected scFvs binding to HER2-ECD in the absence or presence of AL1-PE38KDEL /AL2-PE38KDEL.**

**Supplementary Table S2. Related to Figure 6A. Summary data for 92 HER2-ECD-specific scFvs from the GH2 synthetic antibody library.**

**Supplementary Table S3. Related to Supplementary Figure S3. EC_50_’s for selected scFv-AL1-PE38KDEL binding to HER2-ECD in pH7 and pH5.**

**Supplementary Methods**

**IgG expression/purification**

**References**

**
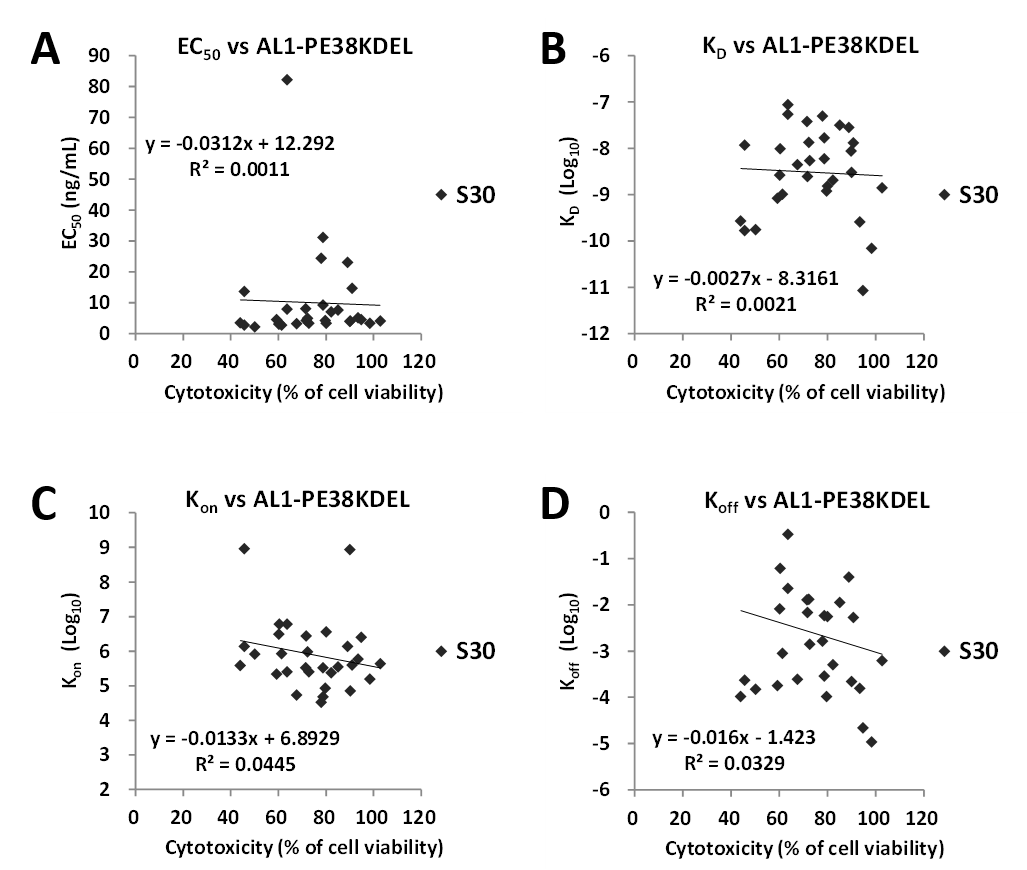
**

**Supplementary Figure S1. EC_50_, K_D_, k_on_, and k_off_ vs. cytotoxicity of scFv-AL1-PE38KDEL immunotoxins.** The EC_50_ (in ng/mL, panel A), K_D_ (in M, panel B), k_on_ (in M^-1^S^-1^, panel C), and k_off_ (in S^-1^, panel D) for the scFvs were plotted against the cytotoxicity of the corresponding scFv-AL1-PE38KDEL immunotoxins. The cytotoxicity measurements are obtained from the data in Figure 6A. The detailed information for these EC_50_ (in ng/mL), K_D_ (in M), k_on_ (in M^-1^S^-1^), and k_off_ (in S^-1^) are shown in Supplementary Table S2.


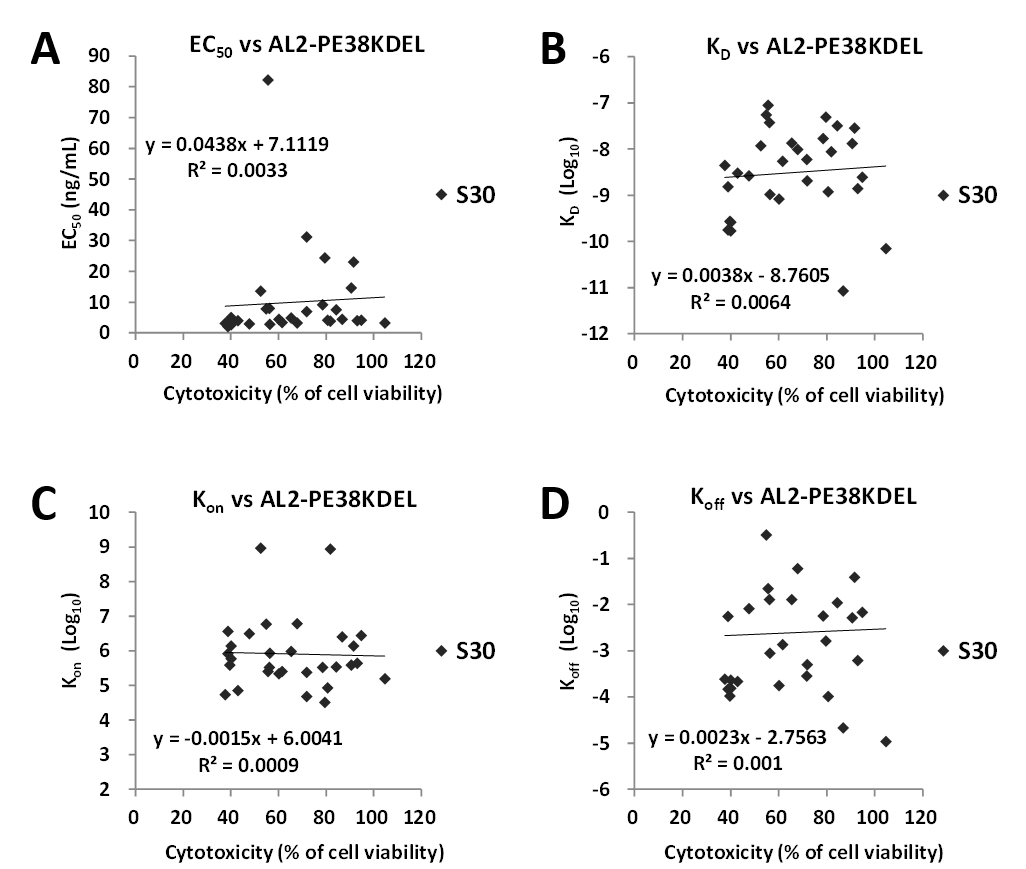


**Supplementary Figure S2. EC_50_, K_D_, k_on_, and k_off_ vs. cytotoxicity of scFv-AL2-PE38KDEL immunotoxins.** The EC_50_ (in ng/mL, panel A), K_D_ (in M, panel B), k_on_ (in M^-1^S^-1^, panel C), and k_off_ (in S^-1^, panel D) for the scFvs were plotted against the cytotoxicity of the corresponding scFv-AL2-PE38KDEL immunotoxins. The cytotoxicity measurements are obtained from the data in Figure 6A. The detailed information for these EC_50_ (in ng/mL), K_D_ (in M), k_on_ (in M^-1^S^-1^), and k_off_ (in S^-1^) are shown in Supplementary Table S2.


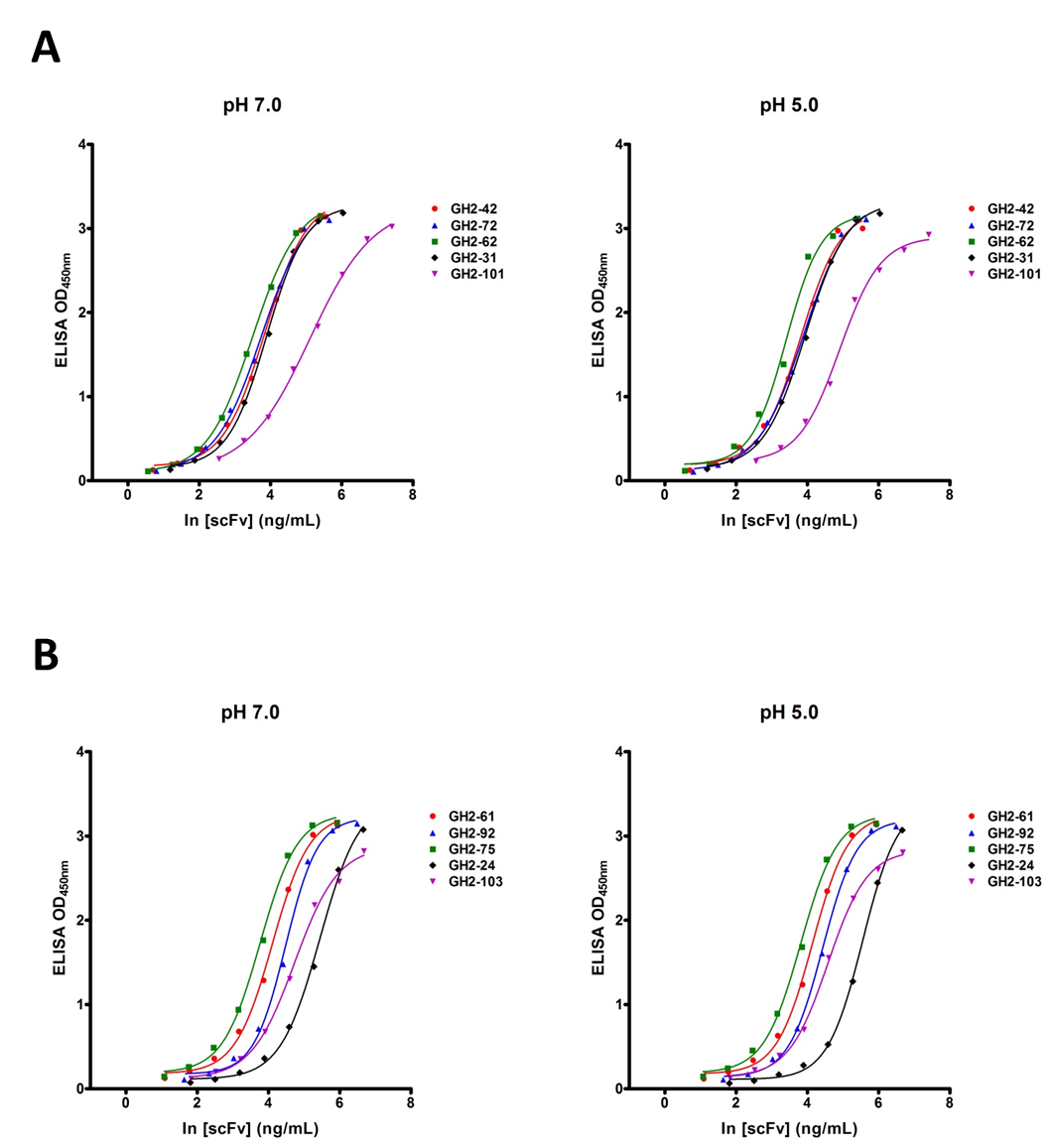


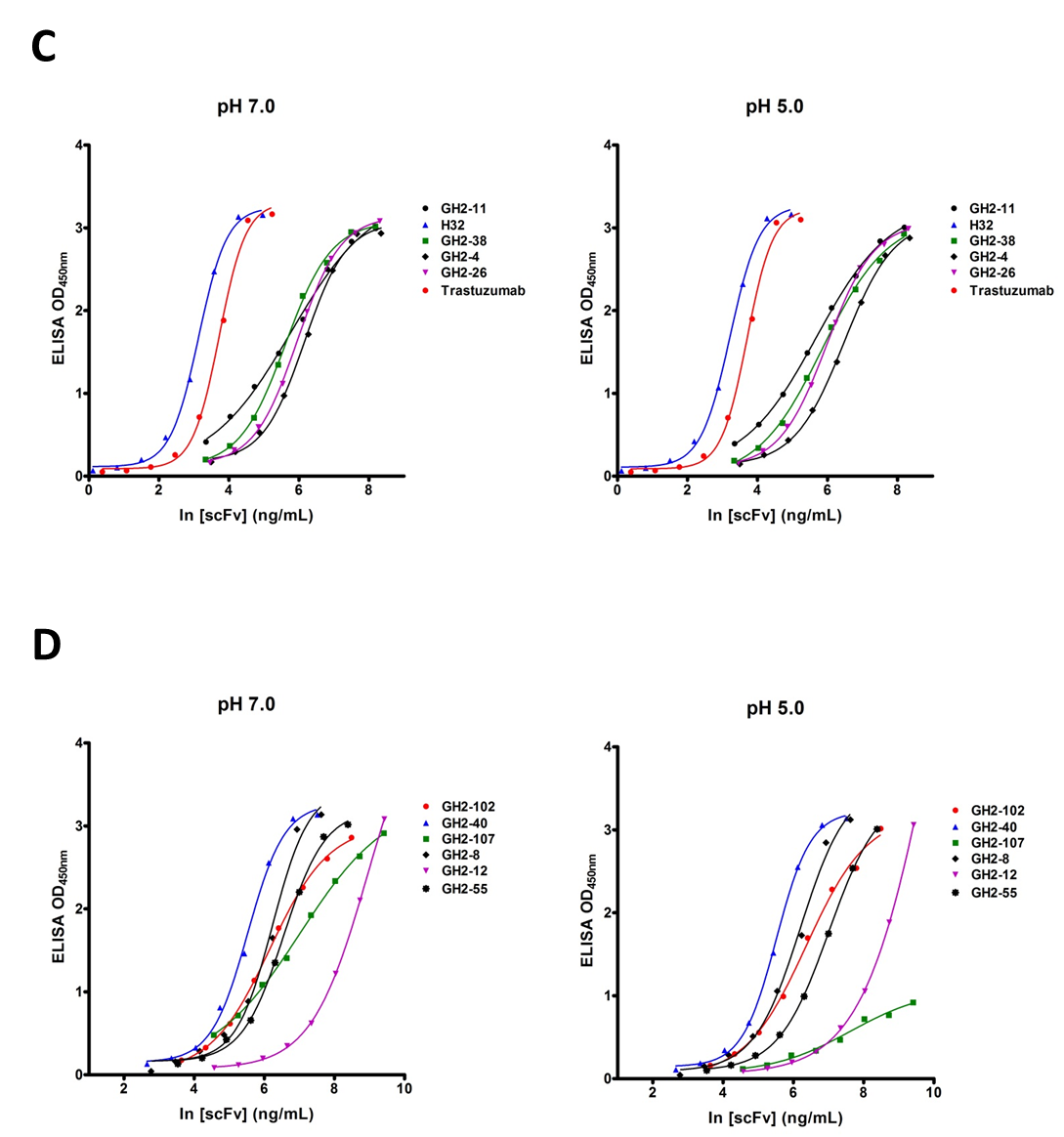


**Supplementary Figure S3. Measurements of EC_50_’s of scFv-AL1-PE38KDEL at pH 7 and pH 5.** (A) scFvs were randomly selected from the top 25% of scFvs in Supplementary Table S2 (ranked in terms of cytotoxicity of the scFv-AL1-PE38KDEL) without histidine in the CDR-H3 sequence. (B) scFvs were randomly selected from the top 25% of scFvs in Supplementary Table S2 with histidine(s) in the CDR-H3 sequence. (C) scFvs were randomly selected from the bottom 25% of scFvs in Supplementary Table S2 (ranked in terms of cytotoxicity of the scFv-AL1-PE38KDEL) without histidine in the CDR-H3 sequence. (D) scFvs were randomly selected from the bottom 25% of scFvs in Supplementary Table S2 with histidine(s) in the CDR-H3 sequence. The EC_50_ measurements and associated sequence information of the scFvs shown in this Figure are summarized in Supplementary Table S3.

**
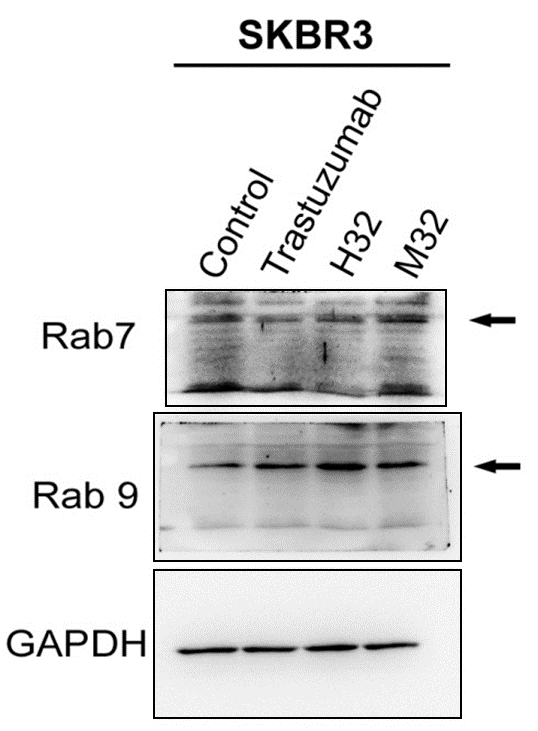
**

**Supplementary Figure S4. Western blot of Rab7 and Rab9 from antibody (IgG) treated SKBR-3 cells.** SKBR-3 cells were respectively treated with trastuzumab (IgG), M32 (IgG) or H32 (IgG) for 1 hr. The full-length Western blot of Rab7, Rab9 and GAPDH from the lysates of the antibody-treated cells are shown in the boxed panels (experimental details are described in Methods). Arrows next to the panels indicate the Rab7 and Rab9 images shown in Figure 8. These Western blots were derived with the same experimental conditions from the same cell lysates.

**Supplementary Table S1. Related to Figure 4. EC_50_’s for selected scFvs binding to HER2-ECD in the absence or presence of AL1-PE38KDEL /AL2-PE38KDEL in pH 7.4.**


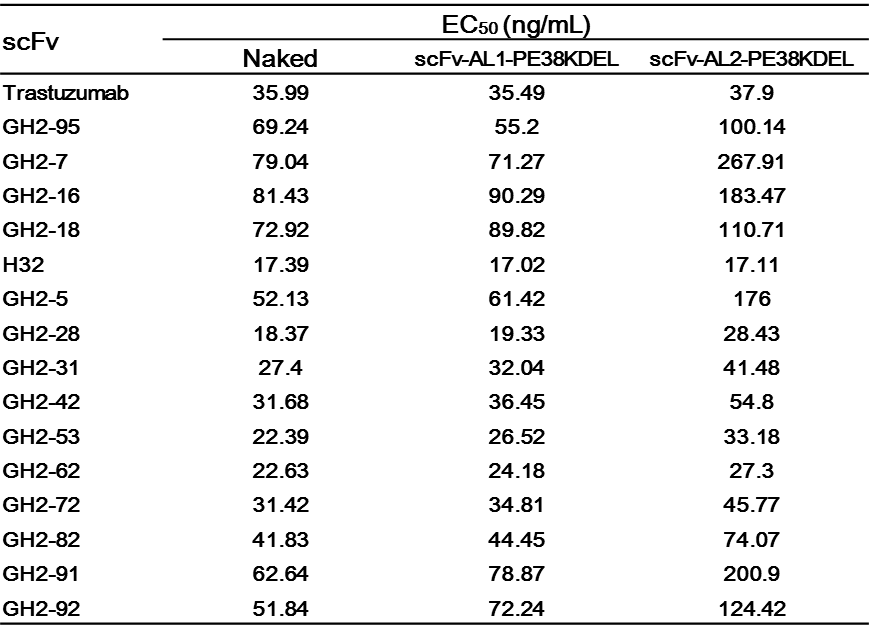


**Supplementary Table S2. Related to Figure 6A. Summary data for 92 HER2-ECD-specific scFvs from the GH2 synthetic antibody library.**

^1^ Epitope group 1 represents the grouping result from the competition assay of 92 GH2 scFv against 6 mouse IgGs (M32, M41, M61, M62, M63, and M64) ^1^; M32-M62 epitope is situated on domains I of HER2-ECD, M41-M61 on domain IV and M63-M64 on domain III ^1^. Epitope group 2 represents the grouping result from the competition assay of 92 GH2 scFv against 5 IgGs of known antibody-antigen complex structures: trastuzumab (epitope: HER2-ECD domain IV), pertuzumab (epitope: HER2-ECD domain II), A21 (epitope: HER2-ECD domain I), Fab37 (epitope: HER2-ECD domain III), and M32 (epitope: HER2-ECD domain I) (see Figure 9 for the composite antibody-antigen complex structures and references therein) ^1^.

^2^ Sequence range of corresponding CDR as indicated by the Kabat numbering ^1^.

^3^ Histidine(s) are colored in red.

^4^ The first, second, third, and fourth quarter of the scFvs are shown in white, light gray, medium gray, and dark gray background, respectively. The ranking order is based on the ranking of the cytotoxicity of the corresponding scFv-AL1-PE38KDEL.

^5^ The data shown for scFvs in the S30 data set (Supplementary Figures S1 and S2), for which the scFvs were randomly selected from the 92 scFvs shown in this Table. The data were measured with BIAcore for the antibodies in human IgG1 form and were reproduced from the previous publication by Chen et al. ^1^.

**Supplementary Table S3. Related to Supplementary Figure S3. EC_50_’s for selected scFv-AL1-PE38KDEL binding to HER2-ECD in pH7 and pH5.**

| **scFv^1^** | **CDR-H3^1^** | **EC_50_ of scFv-AL1-PE38KDEL (ng/mL) ^2^** | |  | **Ratio^2^** |
| --- | --- | --- | --- | --- | --- |
|  | (H96~H100B) | pH 7.0 | pH 5.0 |  | (pH5/pH7) |
| GH2-61 | **YNHHGGV** | 47.12 | 48.70 |  | 1.03 |
| GH2-42 | GFYFDGI | 41.61 | 41.17 |  | 0.99 |
| GH2-72 | FNNDWIG | 37.27 | 37.54 |  | 1.01 |
| GH2-92 | **GNHYDGI** | 77.35 | 77.08 |  | 1.00 |
| GH2-62 | DYLNNGG | 26.25 | 26.18 |  | 1.00 |
| GH2-75 | **DDYHWDG** | 37.79 | 39.61 |  | 1.05 |
| GH2-24 | **GWHNVDN** | 95.95 | 105.53 |  | 1.10 |
| GH2-31 | GNDYDGV | 33.30 | 34.10 |  | 1.02 |
| GH2-101 | YIWFDGL | 95.90 | 98.47 |  | 1.03 |
| GH2-103 | **GHLHDGL** | 65.99 | 58.44 |  | 0.89 |
| GH2-11 | VNWDFYG | 174.01 | 177.91 |  | 1.02 |
| H32 | YYGSRVL | 18.66 | 19.38 |  | 1.04 |
| GH2-102 | **GHHYDGH** | 423.30 | 417.27 |  | 0.99 |
| GH2-40 | **DGHFDGV** | 132.38 | 134.76 |  | 1.02 |
| GH2-107 | **HIHNLWG** | 787.74 | 380874.90 |  | 483.50 |
| GH2-38 | GDNFIGV | 232.35 | 276.80 |  | 1.19 |
| GH2-8 | **GVGYHYY** | 528.30 | 506.42 |  | 0.96 |
| GH2-12 | **YHVYFWW** | 1191.12 | 2106.96 |  | 1.77 |
| GH2-55 | **DIWHNFG** | 352.19 | 476.76 |  | 1.35 |
| GH2-4 | GWNDYDF | 259.81 | 348.50 |  | 1.34 |
| GH2-26 | YFFGDGL | 389.84 | 411.86 |  | 1.06 |
| Trastuzumab | DYFNIGG | 38.95 | 38.65 |  | 0.99 |

^1^scFvs (first column from left) and their corresponding sequence of CDR-H3 (second column from left) are randomly selected from Supplementary Table S2. Histidine residues in the sequences are colored in red. The scFvs in the upper half of the Table (white background) are selected from the first quarter of Supplementary Table S2 (top 25% of scFvs in Supplementary Table S2; ranked by cytotoxicity of the corresponding scFv-AL1-PE38KDEL); the scFvs in the lower half of the Table (grey background) are selected from the fourth quarter of Supplementary Table S2 (bottom 25% of scFvs in Supplementary Table S2; ranked by cytotoxicity of the corresponding scFv-AL1-PE38KDEL).

^2^Ratio=EC_50_(pH5)/EC_50_(pH7). EC_50_(pH5) and EC_50_(pH7) are shown in fourth and third column from left respectively. These EC_50_’s are derived from Supplementary Figure S3.

**Supplementary Methods**

***IgG expression/purification***

*Convert scFv to IgG format:* For IgG expression, the variable domains of light chain (VL) and heavy chain (VH) cDNAs were amplified from the scFv plasmids of binder phages by PCR and then cloned into mammalian expression vector pIgG (a gift from Dr. Tse-Wen Chang, Genomics Research Center of Academia Sinica). The VL domain cDNA was amplified by PCR with proof-reading DNA polymerase (KOD Hot Start DNA polymerase, Novagen) using primer set GH2-VL-F-KpnI (caggtgcacgatgtgat*ggtacc*gatattcaaat gacccagagcccgagcagcctgagc) with GH2-VL-R (TGCAGCCACCGTACGTTTGATTTCCACCTTGGTGCC); for VH domain, using GH2-VH-F (Cgtgtcgcatctgaagtgcagctggtggaatcggga) with GH2-VH-R-NheI (GACCGATGGGCCCTTGGT*gctagc*CGAGCTCACGGTAACAAGGGTGCC). The italic letter of primers indicated the restriction enzyme sites. PCR reactions were performed in a volume of 50 µL with 100 ng DNA template and 1 μL of 10 μM of each primer for 30 cycles (30 sec for 95°C, 30 sec for 56°C, 30 sec for 72°C) followed a 10 min final synthesis step at 72°C. The PCR products were extracted from 1.0 % agarose electrophoresis gel. The linker DNA fragment between V_L_ and V_H_ domains was obtained from pIgG vector by PCR amplification as above, using primer set GH2-IgG-linker-F (AAGGTGGAAATCAAACGTACGGTGGCTGCACCATCTGTC) and GH2-IgG-linker-R (CTGCACTTCAGATGCGACACGCGTAGCAACAGC). The linker fragment includes the constant domain of light chain, bovine growth hormone (BGH) polyA signal, and human cytomegalovirus (CMV) promoter followed by the signal peptide of IgG heavy chain. The above three DNA fragments (VL domain, linker, and VH domain) were assembled by PCR amplification using primer set GH2-VL-F-KpnI and GH2-VH-R-NheI for 30 cycles (30 sec for 95°C, 30 sec for 58°C, 90 sec for 72°C). The PCR products were extracted from 1 % agarose electrophoresis gel and cloned into pIgG vector by Gibson assembly methods^2^. In brief, 2 μL (20 ng) of linearized pIgG vector (digested by *Kpn*I and *Nhe*I previously) and 2 μL (20 ng) insert DNA were mixed with 4 μL Gibson Assembly Master Mix (New England BioLabs Inc. Ipswich, MA, USA) and incubated at 50°C for 1 hour. After then, half volume of ligation mixture was transformed with *Escherichia coli* JM109 competent cells. The DNA insertion of plasmid was confirmed by restriction enzyme digestion and nucleotide sequencing. The constructed vector contains both light chain and heavy chain of IgG, controlled by human cytomegalovirus (CMV) promoter separately.

*Transfection of HEK293 F cells and IgG expression*: For 500 mL culture transfection, suspension 293-F cells in 2-L Erlenmeyer flasks were adjusted to the density of 1.0 x 10^6^ cells/mL. The plasmid DNA (500 μg), diluted in 25 mL serum free medium and sterile with 0.2 μm syringe filter, was mixed vigorously with 25 mL medium containing 1 mg of cationic polymer polyethylenimine (PEI, Polysciences). After 20 min incubation at room temperature, the mixture was added dropwise to the cells with slight shaking, and then the cells were grown in reach-in incubator at 37°C. Tryptone N1 (ST Bio, Inc, Taipei, Taiwan) was added to a final concentration of 0.5% at 24 hr of post-transfection. After 5 days’ culture, the supernatant was collected by centrifugation at 8000 x *g* for 30 min and filtered with 0.8 μm membrane filter (Pall Corporation, Michigan). The supernatant was loaded on HiTrap Protein A affinity column (GE Healthcare, Uppsala, Sweden), and eluted with 0.2 N glycine-HCl at pH 2.50 into 1/10 volume of 1 M Tris-HCl buffer at pH 9.1. The IgG proteins were further purified with Superdex 200 gel filtration column (10/300 GL, GE Healthcare, Uppsala, Sweden) to remove high molecular weight aggregates.

**References:**

1. Chen, H. S. et al. Predominant structural configuration of natural antibody repertoires enables potent antibody responses against protein antigens. *Sci Rep* **5**, 12411 (2015).

2. Gibson, D. G. et al. Enzymatic assembly of DNA molecules up to several hundred kilobases. *Nat Methods* **6**, 343-345 (2009).
